# SARS-CoV-2 Pneumonia: Advances in Diagnosis and Treatment

**DOI:** 10.3390/microorganisms13081791

**Published:** 2025-07-31

**Authors:** Olga Adriana Caliman-Sturdza, Iuliana Soldanescu, Roxana Elena Gheorghita

**Affiliations:** 1 Faculty of Medicine and Biological Sciences, Stefan cel Mare University of Suceava, 720229 Suceava, Romania; olga.caliman-sturdza@usm.ro; 2 Suceava Emergency County Clinical Hospital, 720224 Suceava, Romania; 3 Integrated Center for Research, Development, and Innovation for Advanced Materials, Nanotechnologies, Manufacturing and Control Distributed Systems (MANSiD), Stefan cel Mare University of Suceava, 720229 Suceava, Romania; iuliana.soldanescu@usm.ro

**Keywords:** sequels, COVID-19, therapy, diagnosis, epidemiology

## Abstract

The development of severe SARS-CoV-2 pneumonia is characterized by extensive lung inflammation, which, in turn, leads to respiratory distress and a decline in blood oxygen levels. Hospital admission, along with intensive care or ventilator usage, becomes necessary because this condition leads to serious respiratory problems. This review aims to provide a comprehensive overview of the pathophysiological mechanisms, diagnostic methods, and current therapeutic options for pneumonia caused by the SARS-CoV-2 virus. The pathophysiological process of severe pneumonia due to SARS-CoV-2 infection is characterized by direct lung damage from viral replication, an excessive immune system response, inflammation, impaired gas exchange, and multi-organ failure. The coexistence of various medical conditions leads to substantial lung impairment, resulting in hypoxia and respiratory failure, which can ultimately lead to fatal outcomes. The diagnosis of severe SARS-CoV-2 pneumonia is made through a combination of clinical, radiologic, and laboratory findings. A multifaceted approach integrating antiviral therapy, corticosteroids, oxygen supplementation, ventilatory management, and immunomodulation is imperative to control inflammation and enhance clinical outcomes. Early intervention, meticulous monitoring, and personalized care are paramount for enhancing survival and mitigating complications in critically ill patients with COVID-19 pneumonia.

## 1. Introduction

The SARS-CoV-2 virus causes pneumonia because infection leads to acute respiratory distress and other complications. While most people infected with the SARS-CoV-2 virus have mild symptoms, about 14% develop severe symptoms that require hospitalization due to pneumonia, and 5–10% develop critical symptoms that CoV-2 virus have mild symptoms, about 14% develop severe symptoms that require hospitalization due to pneumonia, and 5–10% develop critical symptoms that require intensive care, including mechanical ventilation due to acute respiratory distress syndrome (ARDS) [1]. The entry of the SARS-CoV-2 virus into the lungs leads to inflammatory responses that create breathing difficulties, which can become fatal [2]. In severe cases of novel coronavirus disease (NCD) (also referred to as severe acute respiratory syndrome (SARS)), caused by the severe acute respiratory syndrome (SARS)-coronavirus-2 (SARS-CoV-2) virus, symptoms include hypoxia, which can progress to acute respiratory distress syndrome (ARDS) or multiple organ failure, requiring medical intervention, including mechanical ventilation and oxygen therapy and sometimes extracorporeal membrane oxygenation (ECMO) [3]. The diagnosis of severe pneumonia due to SARS-CoV-2 infection necessitates a comprehensive evaluation by medical specialists using clinical indicators, as well as X-ray or computed tomography (CT) images, together with polymerase chain reaction (PCR) testing and the analysis of laboratory results to determine both the extent of the lung damage and the severity of the disease [2]. Timely diagnosis and prompt medical intervention are essential for improving outcomes in patients with severe SARS-CoV-2 pneumonia. In this narrative review, we synthesized data from the literature on the pathophysiological mechanisms and diagnostic methods of pneumonia caused by the SARS-CoV-2 virus. Management options for these severe cases of COVID-19 and current therapeutic options were also addressed. The objectives of this study were to systematically extract data on the prognosis and evolution of patients with severe cases of pneumonia caused by the SARS-CoV-2 virus and to highlight the particularities of the management of these cases. The PubMed database and Google Scholar were utilized for the documentation process. The selection of articles was based on keywords such as “COVID-19”, “SARS-CoV-2”, “SARS-CoV-2 pneumonia”, “COVID-19 pneumonia”, “respiratory failure”, and “acute respiratory distress syndrome”. These sources highlight the evidence-based progress made in COVID-19 therapeutics since 2023 and provide an authoritative context for the challenges of immune escape, antiviral resistance, and clinical outcomes in the Omicron era.

## 2. Pathophysiology

The development of severe pneumonia in patients infected with SARS-CoV-2 is driven by a complex interplay of immune responses and virus-induced lung tissue destruction.

**Figure 1:**
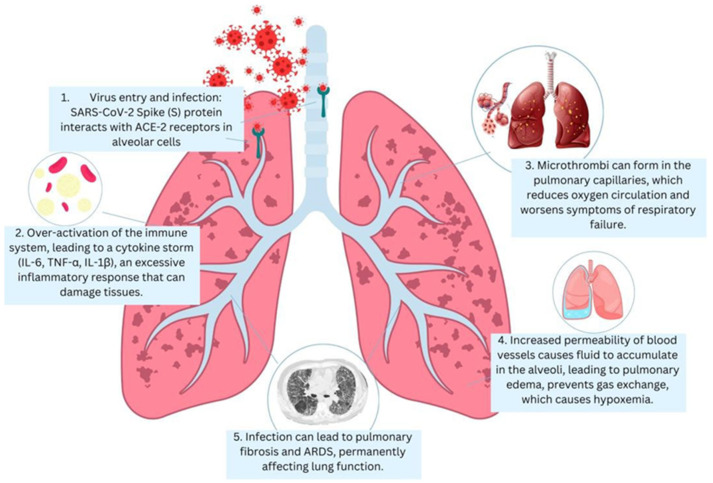
The pathophysiology of severe SARS-CoV-2 pneumonia: 1. Virus entry and infection: SARS-CoV-2 binding to ACE2 receptors in alveolar cells. 2. Immune response: overactivation of the immune system (cytokine storm (IL-6, TNF-α, and IL-1β)). 3. Alveolar damage and edema—increased vascular permeability, leading to fluid buildup. 4. Hypoxemia: impaired gas exchange due to inflammation and microthrombi in pulmonary capillaries. 5. Fibrosis and ARDS: long-term damage, leading to ARDS.

In severely affected patients, SARS-CoV-2 infection of the lower respiratory tract triggers a dysregulated immune response, leading to diffuse alveolar damage, increased alveolar–capillary permeability, and widespread inflammation and coagulopathy [4]. However, although lung pathology constitutes the most frequent data, COVID-19 represents a systemic illness: direct viral damage, hyperinflammation, and endotheliopathy may involve many other organ systems [5]. SARS-CoV-2 can enter the cells of the body with the help of its spike glycoprotein [6]. The S1 subunit of the spike links to the receptor on target cells, angiotensin-converting enzyme 2 (ACE2), and the S2 subunit facilitates viral envelope-to-cell membrane fusion [6,7]. The spike must be cleaved by a host cell protease (TMPRSS2 on respiratory epithelial cells) at the S2 site to activate membrane fusion and permit entry of the viral RNA into the cytoplasm [8]. Within the respiratory system, the virus infects ciliated cells in the nasopharynx and the trachea, which, unable to be contained early by the immune system, can eventually infect the alveoli [9]. The primary target in the lungs is alveolar type II pneumocytes that highly express ACE2 [4]. The functional role of these cells is to secrete the surfactant, but they are also alveolar repair progenitors; thus, their infection and SARS-CoV-2 destruction help undermine the alveolar structure and recovery [10]. The virus then replicates itself once inside the cell, resulting in the formation of double-stranded RNA intermediates, which trigger host defenses against viruses [10]. The stimulation of the innate immune sensing pathways sensitizes SARS-CoV-2 infection and exports interferons and pro-inflammatory cytokines [11]. Pattern recognition receptors identify viral RNA (e.g., RIG-I, MDA5, and endosomal TLRs) and induce the generation of type I/III interferons (IFNs) and tens of interferon-stimulated genes with antiviral effects [12]. This coordinated innate response is responsible for infection in many patients. Nevertheless, the late and dysfunctional early interferon response defines severe COVID-19, when viral replication and antigen loads are maximal [13]. The outcome is a cytokine storm, overenthusiastic immune stimulation with an increase in interleukins (IL-1b and IL-6), tumor necrosis factor (TNF), and other inflammation factors [14]. SARS-CoV-2’s downstream activation of the NLRP3 inflammasome in macrophages results in both the overproduction of IL-18 and cell death (pyroptosis) due to an inflammatory mechanism, which also enhances tissue injury at localized sites [15,16]. The infected lungs are highly infiltrated by neutrophils and monocytes, which liberate proteases, reactive oxygen species, and chemokines, which damage alveolar structures [17]. In the meantime, T cells and natural killer (NK) cells can develop pathological cytotoxic activity, which can lead to immunopathology [18]. In general, there is a high propensity to blunt the adaptive immune response in critical illness, and an exuberant innate inflammatory cascade results in collateral damage to lung tissue and other organs. Diffuse alveolar damage is the diagnostic histopathological feature of severe COVID-19 pneumonia and the prototypical lesions of ARDS [19]. Diffuse alveolar damage (DAD) includes damage to the alveolar epithelium and endothelium in capillaries in large areas; the acute exudative stage is manifested by interstitial edema of the lungs and intra-alveolar edema, a fibrinous hyaline membrane covering alveoli, and pneumocyte necrosis [20]. In COVID-19 autopsies, lungs generally exhibit DAD in exudative and early proliferative stages, which consist of proteinaceous edema, hyaline membranes, massive loss of type I and type II pneumocytes, congested capillaries with microthrombi, and hyperplasia of the surviving type II cells as a failed organization repair attempt [21]. In these pathological alterations, there is a relationship with the clinical picture of ARDS: refractory hypoxemia and poor compliance. The intense leakage of fluid into air spaces results from damage to the alveolar–capillary barrier [10]. Gas exchange is grossly affected by the flooding of alveoli and the development of deposits of hyaline membranes [22,23]. The death of functional type II cells also leads to the loss of surfactant, which supports alveoli collapse (atelectasis) [24]. Some patients with DAD develop fibrotic lung evolution after the acute stage of the disease despite life-saving recovery, which could cause long-term pulmonary fibrosis effects [25]. A notable characteristic of COVID-19 ARDS is severe coagulopathy in the lungs. SARS-CoV-2 infection has been shown to result in the destruction of lung tissue, accompanied by endothelial dysfunction and abnormal clotting, leading to microvascular thrombosis (Figure 1). The development of pulmonary embolism contributes to exacerbated hypoxia, resulting in additional damage to the lungs [26,27]. The presence of signs consistent with disseminated intravascular coagulation (DIC) has been observed in patients, resulting in abnormal blood clotting throughout the body. This has been linked to bleeding complications and thrombosis [28,29]. The pulmonary microcirculation can become pro-thrombotic, which is caused by endothelial injury and inflammatory mediators [30]. Severe COVID-19 cases often have fibrin-rich microthrombi in the small pulmonary arterioles and capillary structures [31]. A damaged endothelium leads to the production of tissue factor and stimulates extrinsic clotting, whereas pro-inflammatory damage liberates intracellular DAMPs, triggering the intrinsic clotting pathway [32]. Platelets aggregate at sites of vascular injury and, together with leukocytes, form intravascular thrombi. Neutrophils also secrete multi-stranded, structured DNA, protein molecules called neutrophil extracellular traps (NETs) that can trap platelets and directly initiate the coagulation process (so-called immunothrombosis) [33]. This yields non-localized fibrin deposition in the lung and clotting factors and platelets. On the clinical level, prevalent D-dimer elevation (a marker of processed fibrin) and thrombocytopenia in patients with severe COVID-19 are associated with poorer outcomes [34]. Early anticoagulation showed lower mortality, and therefore, coagulopathy was the most critical pathophysiology determinant of COVID-19 [35]. Overall, the combination of alveolar edema, inflammatory cell infiltrates, and microvascular thromboses results in an environment known as a pulmonary cytokine storm, leading to ARDS with severe hypoxemia.

Severe pneumonia cases result in immune system overload, which renders patients vulnerable to secondary bacterial or fungal infections, thus exacerbating the existing complications of pneumonia [36]. Systemic inflammation induced by an infection can lead to multi-organ dysfunction, affecting vital organs such as the heart, kidneys, and liver. This arises from the body’s inability to effectively regulate the robust inflammatory response [37].

A substantial proportion of individuals demonstrate resilience to viral infections, often exhibiting recuperation after the cessation of viral replication [38].

SARS-CoV-2 infection can also cause long-term multisystem health challenges even after the acute process of this disease has passed. Such post-acute sequelae of COVID-19 (usually called Long COVID or PASC) include a vast range of malfunctions of multiple organs, including the central nervous system (CNS) and cardiovascular system [39]. Long-term effects are more prevalent in the wake of severe COVID-19, but by now it has become clear that even mild infections may lead to chronic cases of illness or organ damage [40,41]. These long-term effects can extend to months or years and are quite problematic for patients and the health system. The most reported long-term impacts of COVID-19 include neurological and neuropsychiatric sequelae. About 1/3 of all COVID-19 patients who have recovered show some type of neurological or psychiatric disorder even six months after the disease [42]. It is believed that the virus induces both direct viral invasion of the CNS (e.g., through the olfactory bulb or by breakdown of the blood–brain barrier) and a hyperinflammatory state, which causes thromboses and neuroinflammation [40,43,44]. These processes may lead to higher chances of cerebrovascular diseases such as stroke and various CNS signs. Clinically, post-COVID-19 subjects have reported cognitive impairment (or brain fog), as well as memory and concentration problems, headaches, psychiatric problems, sleep disturbance, and even encephalitic syndromes [45]. This observation also raises the fear that the long-term risk of developing diseases such as Alzheimer’s and Parkinson’s may be increased by the acceleration of neurodegenerative processes due to the chronic neuroinflammatory milieu in COVID-19 survivors [46]. Neurological manifestations of Long COVID, hence include not only light cognitive abnormalities but also severe issues, which is why the monitoring of neurological disorders should continue in convalescent patients. Long-term cardiovascular complications have also been reported in COVID-19 survivors. The vasculature and the heart can be infected by SARS-CoV-2, and the ensuing inflammatory and immune responses can cause acute damage to the cardiovascular system, which can result in arrhythmias and acute cardiac injury, as well as myocarditis, thromboembolism, and other cardiac disorders [40]. Some acute cardiac impairments may resolve, but new literature suggests that COVID-19 causes a higher risk of developing new cardiovascular diseases over the long term. Survivors of COVID-19 are more likely to develop new-onset heart diseases—such as ischemic heart disease, heart failure, arrhythmias, and blood clot disorders—compared to those never infected [47]. It is projected that individuals recovering from SARS-CoV-2 infection will experience persistent cardiovascular manifestations, including ongoing myocarditis, vascular dysfunction, and arrhythmias, which could heighten their long-term susceptibility to cardiovascular diseases, like myocardial infarction and stroke [40,47]. These findings indicate that COVID-19 could leave a long-term mark on cardiovascular health and yet require close cardiac monitoring and the treatment of risk factors in patients who have recovered.

The treatment of patients diagnosed with NCD presents a particular challenge when those affected suffer from pre-existing health conditions or exhibit compromised immune systems. In such instances, managing immune responses through therapeutic interventions becomes a particularly challenging aspect of treatment.

## 3. Diagnosis of Severe SARS-CoV-2 Pneumonia

Successfully identifying severe pneumonia caused by SARS-CoV-2 infection requires clinical assessments and laboratory examinations, as well as imaging analysis and microbiological evaluations.

Patients experiencing infection show symptoms that include fever and cough, shortness of breath, and fatigue with muscle aches [48,49]. In cases of acute pneumonia, patients may develop respiratory distress accompanied by chest discomfort, disorientation, and cyanosis [50].

The clinical assessment identifies patients at high risk by evaluating various factors. These include the patient’s age, health problems such as hypertension and diabetes, cardiovascular disease, ongoing respiratory conditions, and a history of contact with viruses [51]. During physical examination, clinicians may detect indications of respiratory distress, including tachypnea and the utilization of accessory muscles to facilitate respiration. Additionally, they may discern unusual lung sounds, such as crackles or wheezes, and observe signs of hypoxia, including a decline in oxygen saturation and the presence of cyanosis [52].

Pulse Oximetry is an early diagnostic instrument used to measure oxygen saturation values [53]. The clinical presentation of severe pneumonia is characterized by oxygen saturation levels that fall below 90%. A diagnosis of respiratory failure severity is determined by the values obtained from Arterial Blood Gas (ABG) testing, which measures hypoxemia levels in conjunction with carbon dioxide retention and acid–base disturbances [54]. The phenomenon of an extremely low arterial oxygenation in COVID-19 patients with normal or near-normal clinical findings is known as happy or silent hypoxia [55,56]. This syndrome is unique to SARS-CoV-2 pneumonia and differs from historic acute airway distress syndrome because most patients have reasonably normal lung compliance and stay alert even with severe levels of hypoxemia [55,57]. Case reports demonstrate that it may cause a sudden worsening and unexpected death because of the delayed identification and treatment of this phenomenon [55,56]. Two principal mechanisms cause happy hypoxia. First, there are blunted chemoreceptive responses: SARS-CoV-2 can cause blunted ventilatory drive secondary to impaired carotid body chemoreceptors and autonomic neuropathy, or perhaps through central hypoxic ventilatory depression (HVD) [56]. The minireview by Kimura suggests central accumulation of adenosine and carotid body dysfunction, and this lowers the respiratory drive, which is partly stimulated by aminophylline [56]. Second, there is ventilation–perfusion mismatch: COVID-19 causes ventilation–perfusion mismatch, shunting, and endothelial damage without affecting lung mechanics, leading to severe hypoxemia but not causing hypercapnia or dyspnea [55,57]. Respiratory alkalosis causes a leftward shift in the oxyhemoglobin dissociation curve, which complicates the interpretation of the pulse oximeter and delays the clinical response. Hypoxemia levels in severe exacerbations are not accompanied by dyspnea, which contributes to underestimating disease severity, subsequent delayed treatment with oxygen, and an increase in care [55] administration, with mechanistic logic and preliminary clinical experience guidance [56]. Further research is needed to delineate the predictors of chemoreceptor dysfunction and the role of pharmacological sensitization of respiratory drive. Clinicians generally begin the assessment process with a chest X-ray examination. In patients with severe pneumonia from SARS-CoV-2, the aforementioned imaging findings may include bilateral infiltrates, consolidation, ground glass opacities, or air bronchograms, which indicate active lung inflammation and structural damage [58]. The disease stage is determined by evaluating whether the X-ray results appear normal. In instances in which X-rays provide no beneficial information, healthcare providers should consider conducting CT scans.

The High-Resolution Chest CT Scan (HRCT) is superior in detecting lung involvement compared to other scans [59]. The diagnostic findings in cases of pneumonia due to novel coronavirus (SARS-CoV-2) include ground glass opacities, which are characterized by inflammation and fluid accumulation. The typical consolidation pattern, characterized by interlobular septal thickening and ground glass opacities, is also present [60,61]. The most serious cases reveal deteriorating pulmonary progression through increasing areas of consolidated tissue in imaging tests. Lung abnormalities, which appear as ground glass opacities in COVID-19 patients, make chest X-rays and CT scans suitable for AI-based screening evaluation.

Convolutional neural networks (CNNs) have been proven to recognize medical patterns in imaging results at a level that equals or surpasses human expert radiologist achievements, especially in deep neural networks [62,63]. Both deep transfer learning model (GoogleNet) and the Residual Network (ResNet-50) are the most effective among the examined methods, according to [64,65]. The ensemble model delivers spectacular outcomes to substantially improve deep learning performance. Multiple computational models, named MultiCOVID, diagnose COVID-19 pneumonia and heart failure, and non-COVID-19 pneumonia and healthy patients by processing both chest X-rays and laboratory tests [66].

The diagnosis of COVID-19 relies on the primary diagnostic test, which involves reverse transcription polymerase chain reaction (RT-PCR) analysis of respiratory samples taken from the nasopharyngeal or oropharyngeal regions [67]. Positive results confirm SARS-CoV-2 infection. Respiratory tract virus levels that are high indicate worse disease outcomes, but these levels differ from patient to patient in terms of symptom severity. The course of severe pneumonia creates conditions that allow bacterial and fungal infections to start. Sputum culture and bronchoalveolar lavage testing help detect extra pathogens and guide medical care when antiviral treatment is insufficient in treating pneumonia cases [68,69].

**Figure 2:**
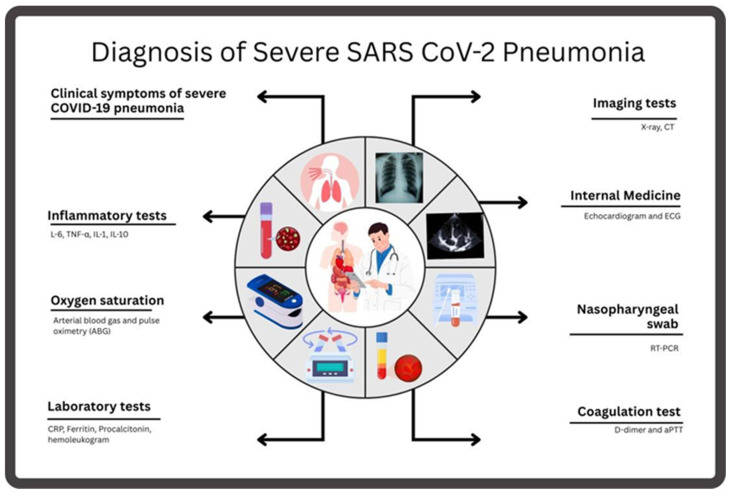
The main assessments used to diagnose SARS-CoV-2 pneumonia include the evaluation of clinical symptoms; a blood test, including a coagulation test; and radiological and inflammatory tests.

If bacteremia is suspected, blood cultures may be obtained to identify secondary bacterial infections. Severe COVID-19 pneumonia leads to elevated white blood cell counts due to inflammation, while some patients display lymphopenia [70]. Elevated C-Reactive Protein (CRP) levels are a marker of systemic inflammation and can be used to monitor disease severity and response to treatment [71,72]. Ferritin measures inflammation, therefore, its levels increase when patients experience severe disease [73]. Procalcitonin may be normal or slightly elevated, and it is used to rule out bacterial coinfection [74] (Figure 2). Elevated D-dimer levels are associated with hypercoagulability and indicate the possibility of microvascular thrombosis or a dangerous condition known as disseminated intravascular coagulation (DIC), which can affect severe COVID-19 cases [75]. The tests evaluate liver and kidney health to determine whether COVID-19 has spread to multiple body organs, since severe COVID-19 cases commonly affect multiple organs.

In instances of severe pneumonia complicated by cardiac involvement, particularly when myocarditis is present, an echocardiogram may facilitate the evaluation of cardiac function. An electrocardiogram (ECG) is indicated for the evaluation of cardiac conditions in patients exhibiting symptoms of arrhythmias or heart-related manifestations.

Medical professionals evaluating SARS-CoV-2 must check for bacterial, fungal, and other viral pneumonia to properly identify COVID-19 pneumonia during testing [76]. Clinicians should test for *Streptococcus pneumoniae*, *Haemophilus influenzae*, influenza, and respiratory syncytial virus, among other possible viral infections.

The classification of severe SARS-CoV-2 pneumonia depends on three main factors, including oxygen need and the extent of respiratory failure, as well as intensive care requirements [77]. For mild pneumonia, administering supplementary oxygen is recommended for minimal periods, provided that the patient does not exhibit signs of respiratory distress. Patients diagnosed with moderate pneumonia require treatment with low-flow oxygen combined with non-invasive ventilation support. The treatment approach for severe pneumonia includes high-flow oxygen therapy and invasive mechanical ventilation or ECMO when combined with ARDS or multi-organ dysfunction symptoms.

## 4. Management of Severe Pneumonia Due to SARS-CoV-2

The management of severe pneumonia due to SARS-CoV-2 infection necessitates a multifaceted approach that emphasizes the following key elements: enhancing oxygenation, regulating viral infection, mitigating inflammation, preventing complications, and supporting organ function [78].

### 4.1. Supportive Care

Adequate oxygenation is the main treatment objective for severe pneumonia management. Oxygen therapy is used to treat hypoxemia, with oxygen saturation level ideally maintained above 90–92% [79]. The delivery of low-flow oxygen via a nasal cannula or simple mask is an appropriate treatment for hypoxemic patients with mild to moderate illness. In patients with moderate to severe pneumonia, health professionals utilize high-flow oxygen in conjunction with non-invasive ventilation (NIV) through continuous positive airway pressure (CPAP) or bilevel positive airway pressure (BiPAP). In cases of severe respiratory failure, mechanical ventilation is imperative [80].

### 4.2. Ventilatory Support

When managing patients with severe COVID-19 pneumonia experiencing respiratory failure, ventilatory support is the primary treatment. The advanced stage of COVID-19 pneumonia commonly triggers ARDS, so healthcare providers need to implement ventilator techniques to balance oxygen delivery management and the avoidance of ventilator-related tissue damage [81,82,83].

### 4.3. Non-Invasive Ventilation (NIV)

A high-flow nasal cannula (HFNC) is often the first line of support for patients with moderate-to-severe hypoxemia [84]. HFNCs have been demonstrated to provide significant oxygen delivery, effectively humidifying the air through nasal cannulas with fewer restrictions than mechanical ventilation. Its application has been demonstrated to reduce respiratory strain and augment oxygen levels, thereby obviating the necessity for endotracheal tube insertion in some cases.

The treatment approach of non-invasive positive-pressure ventilation consists of using NIV systems such as BiPAP or CPAP for patients who have acute respiratory failure to supply positive-pressure ventilation and better oxygenation [85].

The indications for applying non-invasive positive-pressure ventilation include patients with moderate to severe hypoxemia who have a PaO_2_/FiO_2_ ratio of 100 to 200, together with respiratory distress symptoms, elevated breathing effort, tachypnea, or increased oxygen requirements [86]. NIV should be initiated after attempting HFNC when there is evidence of respiratory failure progression despite HFNC use. This includes the following:

(a) Persistent or worsening respiratory distress.
  ◦ Increased breathing effort (e.g., the use of accessory muscles, nasal flaring, or paradoxical breathing).
  ◦ The respiratory rate remains high despite HFNC support.
(b) Worsening gas exchange.
  ◦ SpO_2_ < 90% despite FiO_2_ ≥ 60%.
  ◦ PaO_2_/FiO_2_ (P/F) ratio < 200.
  ◦ Rising PaCO_2_ (hypercapnia) and/or respiratory acidosis (pH < 7.35).
(c) Hemodynamic instability.
  ◦ Hypotension, altered mental status, or signs of organ dysfunction.
(d) Inability to maintain an airway or impending respiratory failure.
  ◦ Fatigue or signs of impending respiratory arrest.

If HFNC fails to stabilize the patient within 1–2 h, transitioning to NIV is recommended to prevent further deterioration and avoid intubation. The implementation of NIV requires active medical supervision. Clinicians must discontinue NIV for patients who develop increasing respiratory difficulty since invasive ventilation becomes the most appropriate treatment.

### 4.4. Invasive Mechanical Ventilation

The medical team is required to use invasive mechanical ventilation (IMV) for patients who fail to respond to NIV treatments or display clinical signs indicating serious deterioration in respiratory status, which includes a PaO_2_/FiO_2_ ratio below 100 and respiratory acidosis with a pH less than 7.25, alongside carbon dioxide retention and non-invasive ventilation failure [87].

The volume-controlled ventilation system is a critical component of the mechanical ventilation process, facilitating the maintenance of set tidal volume ranges through the regulated delivery of pressure. This technique has been demonstrated to help prevent atelectasis formation and ensure consistent ventilation delivery [88]. Pressure-controlled ventilation remains one of the primary choices for controlling plateau pressure because it reduces ventilator-induced lung injury occurrences in ARDS patients [89]. The common ventilator setting, the assist–control (AC) mode, enables the medical device to administer a predetermined number of controlled breaths to patients, in conjunction with an allowance for patient-activated supplementary breaths. Consequently, the respiratory workload is reduced [90].

The primary objective of ventilation treatment is the use of a low tidal volume at a rate of 6 mL/kg of ideal body weight to prevent ventilator-induced lung injury. Applying positive end-expiratory pressure (PEEP) enables healthcare providers to halt alveolar collapse and support lung tissue. A typical PEEP setting for patients with ARDS works best when adjusted with care, since it helps achieve optimal oxygenation while preventing lung over-distension [91]. The acceptance of minor carbon dioxide concentration elevations (hypercapnia) in patients with acute respiratory distress syndrome (ARDS) is appropriate when it decreases both barotrauma and polytrauma risks. It is imperative to maintain lung pressure during plateau at or below 30 cm H_2_O to avert ventilator-associated lung injury (VALI) [92].

### 4.5. Prone Positioning

Medical research demonstrates that patients who suffer from ARDS, including those with COVID-19, can benefit from positioning themselves on their stomachs [93]. This positioning technique facilitates the alignment of ventilation and perfusion, as well as the recruitment of the affected dorsal areas of the lung that are typically affected in ARDS. Prone positioning is a customary practice in mechanically ventilated patients diagnosed with severe ARDS, characterized by a PaO_2_/FiO_2_ ratio below 150. It is recommended that healthcare teams maintain this position for 16 to 18 h daily, if feasible [94]. Medical staff should conduct continuous observation because the process of prone positioning entails considerable effort and introduces significant risks, including potential skin damage, ventilation equipment failure, and respiratory difficulties.

### 4.6. Extracorporeal Membrane Oxygenation (ECMO)

Healthcare providers use ECMO to treat COVID-19 pneumonia patients whose hypoxemia remains unmanageable despite maximal treatment efforts. In ECMO treatment, machines draw blood from the body to oxygenate it before returning the blood to patients, thus enabling the lungs to rest [95]. The indications for ECMO therapy include persistent hypoxemia; a PaO_2_/FiO_2_ ratio below 50 for more than three hours, even with best practices of mechanical ventilation; or multi-organ failure that becomes refractory to standard therapeutic interventions [96]. There is no strict age limit for ECMO, but younger patients (<65 years) generally have better outcomes. Elderly patients (>70 years) have higher mortality, but decisions should be individualized [97]. ECMO is not recommended in frail, multi-organ failure, or end-stage disease patients. In COVID-19, ECMO shows moderate success (40–60% survival) but is less effective when delayed. Initiation after 10 days on IMV was linked to worse outcomes [98]. Obesity, age > 65, and prolonged mechanical ventilation reduced survival [99]. Other studies found that obese patients receiving ECMO for ARDS associated with COVID-19 pneumonia had lower mortality than those without obesity [100]. Consequently, obesity should not be regarded as a contraindication for ECMO. The allocation of ECMO is significantly influenced by resource limitations and ethical considerations. Clinicians have investigated the application of cytokine adsorption devices, CytoSorb and Oxiris, to treat hyperinflammation due to COVID-19 in patients receiving extracorporeal membrane oxygenation (ECMO) therapy through a process known as the “cytokine storm” [101]. No concrete benefits from cytokine adsorption devices such as CytoSorb and Oxiris exist in the current evidence base, although these devices may result in adverse effects. European research, the CYCOV trial, included a single-site investigation of CytoSorb device impacts on severe COVID-19 pneumonia patients needing venovenous ECMO treatment [102]. After a 72 h observation, the study revealed that cytokine adsorption failed to decrease serum interleukin-6 (IL-6) levels and resulted in a decline in 30-day patient survival statistics. The intervention group, which experienced cytokine adsorption, achieved a survival rate of only 18%, while the control group obtained a survival rate of 76%. The Oxiris membrane was evaluated in patients requiring veno-arterial ECMO (VA-ECMO) due to cardiogenic shock. The available literature provides scarce evidence regarding the performance and safety aspects of the Oxiris system when used for COVID-19 ECMO treatment [103].

### 4.7. Ventilator-Associated Lung Injury (VILI) Prevention

It is recommended that the minimum possible tidal volumes be set to 6 mL/kg of one’s ideal body weight to prevent VILI among ARDS patients. Maintaining safe mechanical ventilation requires appropriate oxygen level management, with saturations maintained at 88–95%, and PaO_2_ should stay between 55 and 80 mmHg (Table 1) [104].

### 4.8. Weaning and Extubation

Weaning from mechanical ventilation involves attaining SpO_2_ levels exceeding 90%, necessitating minimal PEEP, augmented respiratory effort, and adherence to fundamental directives from healthcare professionals. Medical staff utilize SBTs by transitioning patients from ventilator support to CPAP or pressure support ventilation to ascertain readiness for returning to spontaneous breathing [105]. The removal of the breathing tube from the patient is contingent upon the confirmation of two things: first, the patient’s ability to maintain proper oxygenation levels, and second, the patient’s ability to maintain ventilation functions.

### 4.9. Ventilatory Support for Specific Populations

Healthcare providers need to carefully fine-tune ventilator settings, particularly PEEP and tidal volume, for obese patients, since they face an increased risk of severe COVID-19 due to their obesity status [106]. Elderly patients and other vulnerable groups require ventilatory support for extended periods, during which healthcare professionals monitor the development of ventilator-associated pneumonia and other complications [107].

Healthcare providers require proper personal protective equipment (PPE) to guard against viral transmission because aerosol-generating procedures, such as intubation and NIV, are highly risky.

To overcome oxygen supply limitations and avoid ventilator-related complications, novel micro- and nanostructured systems are being investigated. These include oxygen-loaded microbubbles and nanobubbles, as well as nanocarrier-based oxygen delivery through perfluorocarbon-based emulsions and hemoglobin-based oxygen carriers (HBOCs) in the form of liposomes or other polymeric nanoparticles. These carriers can supply blood-dissolved oxygen and support gas exchange, even when the mechanical ventilator is not operating, and can serve as a potentially portable, scalable oxygenation strategy in moderate or acute COVID-19 respiratory failure [108]. Nanotechnology presents the possibility of creating multifunctional strategies, such as oxygen delivery with therapeutic and diagnostic options. Nanocapsules loaded with hemoglobin or perfluorocarbon nanoemulsions can increase tissue oxygenation and can also be delivered, targeted, and fully recirculate [108]. The combination of these systems with either anti-inflammatory or antiviral systems in a hybrid nanomedicine platform could help target both hypoxemia and pathophysiology. However, despite its preclinical status, such nanostructured oxygen carriers could direct more activities to alleviating the burden on intensive care and enhancing clinical outcomes in hypoxemic COVID-19-infected individuals by either adding to or even replacing conventional oxygen treatment.

### 4.10. Antiviral Treatments

The Food and Drug Administration (FDA) has authorized the emergency use of remdesivir for patients experiencing severe cases of SARS-CoV-2 infection while receiving hospital care. The mechanism of action of the drug involves the inhibition of viral RNA-dependent RNA polymerase, thereby preventing viral replication [109]. It is typically administered intravenously for up to 5 to 10 days, and it starts with a loading dose (200 mg on day 1), followed by 100 mg daily for the next 4 days. During treatment, the pharmaceutical dosages are adjusted according to each patient’s specific circumstances [110]. Remdesivir is most commonly administered to patients who are hospitalized due to severe COVID-19 pneumonia and require supplemental oxygen. Its 423 use is typically recommended for patients with moderate to severe disease, though its 424 efficacy is not as pronounced in patients with extremely severe symptoms who require 425 high levels of oxygen or ventilation support [111]. Available clinical evidence shows that 426 remdesivir reduces the recovery duration for patients with severe COVID-19 pneumonia 427 yet has a limited relationship to patient survival rates [112]. When administered early in the disease process, remdesivir achieves its best therapeutic outcomes, but it should be given before a patient reaches critical illness [113]. Initiating remdesivir therapy during hospitalization within the first seven days of symptom development supports accelerated recovery and a decrease in mortality according to studies, including the ACTT-1 trial and subsequent research [114]. The antiviral activity of remdesivir continues to affect all Omicron variant branches, along with its subvariants. The highly different XBB.1.5 and BA.2.86 strains demonstrate comparable remdesivir susceptibility levels to the original virus strain, according to laboratory results [115]. Infection with viruses that demonstrate remdesivir resistance remains unusually rare because immunosuppressed patients treated with extended therapy rarely develop remdesivir-resistance-causing polymerase mutations, which typically fail to persist due to negative impacts on virus fitness [116]. The U.S. FDA introduced a crucial expansion in 2023 regarding remdesivir treatment by giving its approval for patients with severe kidney disease and patients undergoing dialysis based on studies demonstrating safety in these groups. The development of outpatient medication GS-5245, also known as obeldesivir, serving as the oral analog of remdesivir, failed to reach its clinical trial goals [117]. Tomsgarosivir failed to enhance symptom resolution in non-hospitalized patients during Phase 3 trials, since most participants had excellent recovery times, even on placebo medication. High levels of pre-existing immunity among patients caused the illness duration to decrease, thus making it difficult to observe any additional helpful effects of the drug. The main side effects of remdesivir were minor increases in liver enzyme measurements [118]. Medical tests should also measure renal function, because kidney problems have developed in some patients’ post-treatment [119].

Nirmatrelvir/ritonavir (Paxlovid) is an oral antiviral therapy used in mild-to-moderate disease and may be considered in some hospitalized patients based on clinical judgment and the timing of infection [108]. The mechanism of action of nirmatrelvir/ritonavir involves the inhibition of the protease enzyme that is essential for the viral replication process. The medication is formulated as an oral dosage form demonstrated to be effective in reducing viral replication. It is important to note that nirmatrelvir/ritonavir medications can result in changes in taste perception, as well as diarrhea, and may affect drug interactions through its ritonavir component [120]. Research conducted through the EPIC-HR trial (during the Delta wave) demonstrated that nirmatrelvir/ritonavir decreased the hospitalization risk and death rate of unvaccinated high-risk patients by approximately 89% [121]. An evaluation of nirmatrelvir/ritonavir performance in actual patients shows the medication remains effective but provides reduced hospitalization prevention results among vaccinated individuals. Nirmatrelvir/ritonavir maintains its effectiveness against all Omicron subvariants because the protease, which is its target, remains very well preserved [122]. Laboratory experiments prove that nirmatrelvir chemically inhibits BQ.1.1 alongside XBB.1.5 and BA.2.86 variants at the same intensity as previous strains [123]. Two ongoing concerns related to the clinical use of nirmatrelvir/ritonavir include viral rebound in select patients and the need for drug interaction assessment because ritonavir inhibits CYP3A pathways. Hence, patient medication review remains critical. The emergence of nirmatrelvir drug resistance remains confined and limited because the virus loses fitness and its protease mutations revert to the wild type after the treatment of immunocompromised patients [124]. Stewardship practices should be sustained through the selective distribution of nirmatrelvir/ritonavir to patients 473 who will receive the greatest treatment benefits.

An oral prodrug of a ribonucleoside analog, molnupiravir, induces lethal mutagenesis in the viral RNA genome. This drug received authorization for treating high-risk outpatients who have no other acceptable options. Molnupiravir can combat all current variants because it attacks the viral polymerase instead of spike proteins, but its DNA-altering mechanism raises concerns regarding the future development of antiviral resistance. Rebellion seems to be molnupiravir’s mutagenic approach, because it generates specific mutation patterns in SARS-CoV-2 viral populations, as research in 2023 demonstrated [125]. Recent Omicron lineage viruses with mutations linked to molnupiravir appeared through global sequencing data, suggesting that the drug possibly stimulates the evolution of new viral strains. Research confirms that drug-produced variants from patients do not develop more dangerous properties or spread extensively throughout the population [126].

High-risk patients under medical guidance can use molnupiravir orally during the beginning of their illness, but the medicine typically remains limited to moderate-illness and pediatric cases (Table 2) [127].

Shionogi created ensitrelvir (Xocova), a new oral 3CL protease inhibitor that reached the market after 2023 [135]. The Japanese authorities approved Xocova for emergency use in 2022 and granted full approval in 2023. Ensitrelvir does not require ritonavir to boost efficacy; thus, it reduces the complexity of treatment administration. The Phase 3 SCORPIO-SR clinical trial conducted ensitrelvir assessments on vaccinated and low-risk patients during the BA.2 Omicron wave in Asian regions [135]. Patients treated with ensitrelvir (125 mg daily for 5 days) experienced a reduction in their COVID-19 symptoms much faster than placebo-based treatment (by 24 h) according to a published study [132]. Tests measuring viral loads showed that ensitrelvir treatment leads to a more rapid reduction in virus levels in patients compared to their untreated peers. The efficacy trial data demonstrated that ensitrelvir provides benefits across vaccinated healthy people by reducing the illness duration because 91% of participants received at least two vaccine doses in addition to having no severe disease risk factors. Testing of ensitrelvir occurred in unvaccinated, high-risk populations, whereas nirmatrelvir/ritonavir and molnupiravir received exclusive testing in this population group. Studies show that ensitrelvir exhibits secure properties, as its administration led to adverse events comparable to a placebo in clinical trials. The protease-targeting capacity of ensitrelvir matches nirmatrelvir since it attacks the protease sequence that stays constant. Thus, it remains effective against current Omicron subvariants [131]. Further international approval of ensitrelvir would enhance the antiviral toolkit because it can treat patients who cannot take nirmatrelvir/ritonavir because of drug interactions or because of the need for outpatient treatment.

The field expanded after 2023 due to several new treatment options. The pharmaceutical company Pfizer is currently developing ibuzatrelvir as a sole oral protease inhibitor without ritonavir requirements. Clinical research on ibuzatrelvir treatment for standard-risk adults during Phase 2b trials demonstrated strong antiviral effects (≈0.7–0.8 log_10_ viral load reduction versus placebo by day 5) together with positive safety results [135]. Researchers found that every dose of ibuzatrelvir decreased SARS-CoV-2 RNA levels, and its effects were supported by positive clinical trial results.

Chinese scientists conducted research on VV116 (JT001), a remdesivir analog, along with other oral nucleoside analogs. During the Omicron BA.5 trial, VV116 demonstrated equal effectiveness as nirmatrelvir/ritonavir in achieving clinical recovery while avoiding many drug-related side effects [130]. Soon after its initial publication in 2023, the Chinese authorities included VV116 in their formal drug approval list. A single subcutaneous peg-IFN-λ injection proved effective as an outpatient treatment according to Phase 3 TOGETHER trial results, which demonstrated a 50% reduction in hospital admittance (4% vs. 6% for a placebo) [129]. This efficacy was found for people with different strains of COVID-19 (Delta and Omicron), and vaccinated patients also benefited from the drug. The antiviral defenses in the respiratory mucosa receive a boost from IFN-λ because this immunomodulatory protein functions against host cells and not the virus, and therefore exhibits universal variant compatibility. The publication of positive results in NEJM this year has not led to timely regulatory acceptance of the treatment. The Food and Drug Administration denied an emergency authorization request because the study took place in foreign facilities and because pandemic conditions had evolved. Thus, interferon lambda is a novel therapeutic agent that shows promise for future viral outbreaks because it maintains effectiveness against highly mutated viral strains (Table 3).

### 4.11. Corticosteroids

Dexamethasone is the standard corticosteroid medication when treating severe COVID-19 pneumonia in patients [136] and is recommended for patients requiring supplemental oxygen or invasive ventilation. Dexamethasone controls widespread inflammation throughout the body, as it prevents the excessive immune response that leads to worse lung trauma during a cytokine storm. Typically, it is administered at a dose of 6 mg once daily for up to 10 days [137]. In specific clinical circumstances, both methylprednisolone and other forms of steroids can be used, but dexamethasone maintains its position as the treatment standard [138]. Large clinical trials, including RECOVERY, have proven that dexamethasone effectively lowers the mortality rate among COVID-19 patients who need supplemental oxygen or mechanical ventilation [139]. Remdesivir is most effective in the early stages of severe disease, but is still used in hospitalized patients who require supplemental oxygen. The effectiveness of dexamethasone becomes apparent during disease progression, particularly when inflammation, coupled with an overactive immune system, becomes a major concern. Dexamethasone creates several unwanted side effects that become notable when using it for an extended period, including elevated blood glucose levels, water retention, and elevated susceptibility to infections [140]. The primary benefits associated with the utilization of these drugs are typically observed during the brief treatment intervals used minimize the potential of deleterious side effects.

### 4.12. Anti-Inflammatory and Immunomodulatory Therapies

Tocilizumab, an anti-inflammatory medication, functions as an IL-6 inhibitor. It can be administered to patients with severe cases of pneumonia caused by the novel coronavirus who exhibit elevated concentrations of IL-6 and significant systemic inflammation, as indicated by elevated CRP or ferritin levels [141]. The medication contributes to minimizing high levels of inflammation, which occurs during cytokine storm situations. Its use in conjunction with steroid medications has been shown to reduce mortality. However, it is imperative to note that tocilizumab administration has been associated with an elevated risk of infection, hepatic complications, and a decline in white blood cell counts [142]. Additional studies are needed to establish the efficacy of tocilizumab when administered at different stages of SARS-CoV-2 infection, as well as the safety of its administration in children under the age of 18.

The Janus kinase inhibitor baricitinib functions as an immunomodulatory drug, which healthcare providers use in severe COVID-19 patients, together with dexamethasone [143]. Anakinra, an IL-1 receptor antagonist, may also be considered for patients with severe disease who have signs of systemic inflammation [144]. Another IL-6 inhibitor is sarilumab, and tofacitinib can also be used as a JAK inhibitor. Using either IL-6 inhibitors or JAK inhibitors (baricitinib preferred over tofacitinib) is recommended in patients with elevated inflammatory markers. Studies have not shown any benefit of the use of lopinavir/ritonavir, hydroxychloroquine, azithromycin, ivermectin, or convalescent plasma in patients with severe COVID-19 pneumonia. Earlier in the pandemic, Sotrovimab and Bamlanivimab/Etesevimab monoclonal antibodies received extensive use, but current variants have reduced their clinical impact. The binding mechanism of monoclonal antibodies targets the virus spike protein to block cellular entry, thus stopping virus replication during the early stages of infection [145]. Monoclonal antibodies have maximum effectiveness when given during the first week of infection to high-risk patients who remain out of the hospital. For hospitalized patients with severe pneumonia, their treatment benefits are restricted.

Vilobelimab is a novel anti-C5a monoclonal antibody that can reduce immune system activation and inhibit lung injury, being effective in patients with ARDS and multiple organ failure due to severe or critical COVID-19 [146,147,148]. Vilobelimab has been shown to specifically bind to C5a, thereby reducing the inflammatory response without compromising the immune system’s overall functionality. It is important to note that vilobelimab is currently recommended solely in the context of a clinical trial. Further studies are necessary to investigate the effects of this monoclonal antibody when administered after 48 h of intubation. Moreover, further studies are necessary to ascertain its efficacy and toxicity when administered concomitantly with baricitabine or other immunomodulators. New studies are needed to establish exactly which patient groups might benefit from vilobelimab treatment, as well as its safety in 601 immunocompromised people [149,150,151,152,153,154]. Researchers have since identified new antibodies 602 with even broader activity—including some bispecific antibodies that bind two epitopes 603 simultaneously to prevent escape [155]. A leading contender is VYD222 (commercial name pending, by Invivyd), a monoclonal antibody engineered to target a conserved region of the Omicron spike RBD [156]. In early 2023, in vitro studies showed that VYD222 neutralizes all tested Omicron subvariants, including XBB.1.5 and the BA.2.86 lineage, at clinically achievable concentrations. It also has a half-life extension modification to provide long-lasting protection. Invivyd initiated the Phase 3 CANOPY trial of VYD222 as a prophylaxis in immunocompromised patients. By late 2023, exploratory Phase 1 data indicated high neutralizing titers in human recipients, and the company submitted an EUA request to the FDA for VYD222 as a preventive therapy.

### 4.13. Antibiotics and Antifungals

Secondary bacterial infections triggered by COVID-19 develop most frequently among people who suffer from severe pneumonia, along with prolonged stays in ICU settings [157]. Clinicians frequently initiate broad-spectrum antibiotic therapy, which includes medications such as ceftriaxone, piperacillin–tazobactam, and meropenem before bacterial culture results are available [158]. The levels of procalcitonin and CRP in the bloodstream assist medical professionals in distinguishing between bacterial and viral infections, thus leading to correct antibiotic therapy choices [159]. Immunocompromised patients are prone to secondary infections from fungi such as *Aspergillus* or *Candida*, which require antifungal treatment.

Patients requiring IMV or ECMO with severe immunosuppression, such as neutropenia, hematologic malignancies, organ transplant, or prolonged corticosteroid use, are at a high risk of invasive pulmonary aspergillosis (IPA) [160]. COVID-19-associated pulmonary aspergillosis (CAPA) can be caused by immune dysregulation or disruption of mucociliary clearance [161]. Other risk factors include prolonged ICU stay, prolonged ventilator dependence, the use of broad-spectrum antibiotics, and the use of high-dose steroids [162]. Prolonged antibiotic use leads to microbiome imbalance, which can lead to invasive fungal infections. Anti-IL-6 therapies (tocilizumab) or JAK inhibitors increase fungal susceptibility [163]. The galactomannan (GM) antigen test is a diagnostic tool used for invasive aspergillosis, detecting *Aspergillus* cell wall components in serum or bronchoalveolar lavage (BAL). The GM test is non-invasive, with high sensitivity and specificity (especially in BAL fluid), but with lower sensitivity in non-neutropenic ICU patients, such as those with COVID-19 and ECMO. The serum GM test may be negative in localized lung infection, so BAL GM testing is more reliable than the serum in ventilated or ECMO patients. New molecular tests employing PCR and next-generation sequencing (NGS) can detect bacterial, viral, and fungal DNA/RNA in a matter of hours, providing crucial guidance for administering antimicrobial therapy [164]. These rapid molecular tests are more efficient than traditional culture methods in detecting difficult-to-culture pathogens, such as *Aspergillus*, *Legionella*, and *Mycoplasma*. Multiplex panels can detect multiple pathogens simultaneously (e.g., pneumonia panels detecting bacterial and viral co-infections) and facilitate the de-escalation of broad-spectrum antibiotics. However, it is important to note that these tests do not differentiate between colonization and active infection, and they may produce false-negative results in cases of low pathogen loads. Additionally, these tests are costly and not widely available in all medical facilities [165]. Rapid PCR tests are especially useful for critically ill patients with conditions such as ventilator-associated pneumonia, sepsis, or suspected fungal infections such as CAPA.

### 4.14. Management of Coagulation Abnormalities

Severe cases of COVID-19 infection lead to an increased risk of blood clot development and DIC. For hospitalized patients, low-molecular-weight heparin and fondaparinux are recommended anticoagulant medications, notwithstanding any contraindications [166]. Therapeutic anticoagulation, which includes therapeutic heparin, is appropriate when patients have confirmed venous thromboembolism (VTE) or DIC [167]. The management of coagulation abnormalities in severe COVID-19 pneumonia is a critical aspect of patient care, as COVID-19 is associated with an increased risk of thrombotic events, including deep vein thrombosis (DVT), pulmonary embolism (PE), and DIC [168]. Disease-related complications have been demonstrated to increase the overall mortality and morbidity associated with this condition. Elevated D-dimer levels are a common finding in severe cases and are often used as an indirect marker of clot formation and fibrinolysis. Consequently, patients with elevated D-dimer levels are predisposed to thrombotic complications [169]. The prothrombin time (PT) and International Normalized Ratio (INR) can be used to monitor the extrinsic coagulation pathway and liver function, especially in cases of DIC. Thrombocytopenia commonly appears in COVID-19 infections, either to mark disease severity or to indicate active DIC. The Activated Partial Thromboplastin Time (aPTT) assessment method allows clinicians to monitor for both clotting factor deficiencies and possible cases of DIC [170].

Most acutely hospitalized COVID-19 patients receive low-dose anticoagulation through low-molecular-weight heparin (LMWH) or unfractionated heparin for protecting themselves from VTE, even if their disease severity is mild to moderate [171]. ICU patients, those with severe pneumonia, and patients receiving mechanical ventilation may achieve better outcomes through therapeutic applications of unfractionated heparin and LMWH at higher doses.

DIC is characterized by microvascular clot formation, which disseminates throughout different parts of the body following severe infection with the SARS-CoV-2 virus. Managing DIC involves the administration of supportive care for the underlying infection in conjunction with the utilization of direct antiviral medications and corticosteroid treatment for cases of COVID-19. In cases where DIC results in significant bleeding complications accompanied by abnormal coagulation, patients require the administration of fresh frozen plasma (FFP) and platelets [172]. LMWH, alongside unfractionated heparins, is the primary treatment for confirmed PE in patients suffering from COVID-19. The functional capacity of platelets remains impaired in cytokine storms, even if patient blood tests show normal platelet counts [173].

A cytokine storm occurring during COVID-19 infection leads to major procoagulant activation within the body. Corticosteroids, along with tocilizumab, act as anti-inflammatory medications that minimize both inflammatory reactions and coagulation abnormalities [174]. Clinicians can evaluate coagulation risks and thrombosis hazards according to physical virus quantities along with measurements of CRP, ferritin, and IL-6 markers [175]. Striking a proper balance between anticoagulation therapy requirements should include consideration of bleeding hazards, especially in critical patients and individuals presenting liver dysfunction or gastrointestinal bleeding [176]. Close monitoring of the patient’s clinical status and coagulation parameters is essential. Extended thromboprophylaxis may be considered in patients with severe disease who are discharged, especially those with known thrombotic risk factors such as previous VTE, prolonged immobilization, obesity, or advanced age. The required medications for anticoagulation therapy after discharge include LMWH or direct oral anticoagulants (DOACs) that must be taken for multiple weeks [177].

Therapy selection should depend on a patient’s medical status combined with their individual thrombosis risk factors, in addition to their reaction to the prescribed treatment. The optimal management strategy necessitates collaborative efforts among intensivists, hematologists, and other specialized professionals.

### 4.15. Monitoring and Supportive Measures

The management of fluids requires special attention in patients with ARDS because additional fluids may lead to increased pulmonary edema [178]. Hydration therapy needs to be kept in equilibrium to preserve adequate blood flow but prevent the deterioration of respiratory conditions. The regular assessment of renal and liver functions becomes essential for COVID-19 pneumonia severity because patients can develop combined organ dysfunction [179]. The medical requirement to deliver enteral or parenteral nutrition sustains patients on mechanical ventilation because they need appropriate nutritional care for their recovery.

### 4.16. Sedation, Analgesia, and Psychosocial Support

Patients requiring invasive ventilation may require sedation to ensure comfort, prevent agitation, and synchronize with the ventilator. The main medicines used for sedation include fentanyl in addition to propofol and midazolam [180]. Patients in critical care settings who need mechanical ventilator support benefit from mental well-being support for their anxiety symptoms and delirium, as well as feelings of social isolation.

### 4.17. Preventing Complications and Secondary Infections

The prevention of infections acquired in hospitals (e.g., ventilator-associated pneumonia and catheter-associated infections) is a top priority of the rigorous control of infections, including hand hygiene procedures and isolation requirements [181]. The long-term management of patients with long-term sequelae of the disease (long-term disease management) depends on the administration of the vaccine for the disease caused by COVID-19. This measure is crucial to control the severity of the disease and its transmission.

### 4.18. Rehabilitation and Long-Term Care

Pulmonary rehabilitation is a treatment that healthcare professionals need to provide to patients who have had prolonged stays in the ICU or required mechanical ventilation, because they need to rebuild their lung function and exercise capacity.

Long-term symptoms associated with the novel coronavirus, referred to as LongCOVID-19, have been observed in a significant proportion of patients, manifesting in symptoms such as fatigue, dyspnea, and cognitive dysfunction [182]. To address these challenges, a collaborative approach among healthcare specialists is imperative, with a focus on both managing post-hospital syndromes and enhancing healing processes.

### 4.19. Clinical and Treatment Features of COVID-19 Pneumonia Due to the Omicron Strain

The clinical and treatment features of the Omicron variant of SARS-CoV-2 differ from previous variants in terms of transmissibility, severity, and response to treatment. The Omicron variant exhibits a higher degree of transmissibility compared to earlier variants, including the Delta variant. It has a shorter incubation period, ranging from 2 to 4 days after exposure, and a milder disease severity compared to the Delta variant [183]. More cases of Omicron infections are asymptomatic or mild and have a lower risk of hospitalization and severe disease, especially in vaccinated individuals [184].

The Omicron variant has less of an impact on the lungs compared to previous strains, reducing the risk of severe pneumonia and ARDS [183]. Common symptoms include a sore throat, nasal congestion, runny nose, sneezing, headache, fatigue and muscle aches, fever (less common than with Delta), and coughing (usually dry). A loss of taste/smell is less common than with Alpha/Delta variants. Gastrointestinal symptoms (nausea and diarrhea) can occur but are less common. Severe pneumonia and ARDS are rare and are mainly found in high-risk groups. The continual emergence of Omicron sublineages since 2023 has tested the resilience of COVID-19 treatments. Each major subvariant—XBB.1.5, BQ.1.1, BA.2.86, JN.1, and others—carries unique constellations of spike mutations that challenge immunity and therapeutics [129]. Treatment features for Omicron infection in mild cases consist of rest, hydration, and symptom management (e.g., acetaminophen for fever and pain). Antiviral medications such as nirmatrelvir/ritonavir are effective in reducing severity, especially in high-risk patients [184]. Molnupiravir is an alternative for those ineligible for nirmatrelvir/ritonavir, and remdesivir is indicated for hospitalized patients. The characteristics that define Omicron subvariants include immune escape, which allows the viruses to bypass antibodies that derive from previous infections, vaccinations, and therapeutic mAbs. The SARS-CoV-2 variant XBB.1.5 (also known as “Kraken”) emerged from two BA.2 descendant sequences through a recombination event with numerous RBD mutations [185]. The strain displayed the most significant immune evasion properties by effectively evading antibodies produced by vaccines [186]. Laboratory results showed that XBB.1.5 and its related strains, XBB.1 and XBB.1.16, nearly completely escaped the neutralizing antibodies of triple-vaccinated individuals [129]. Many monoclonal antibodies used for Delta and earlier strains lost effectiveness against Omicron subvariants [187]. Therapeutic monoclonal antibodies have to be continually reformulated to keep pace with these subvariants, but there was a period in 2023–2024 with none available. Patients at risk had to rely on small-molecule antivirals and boost their vaccine doses for protection. The emergence of JN.1 and BA.2.86 has been a key motivator for developing variant-proof antibodies (as discussed with VYD222) [188]. The development of new Omicron subvariants did not cause significant harm to small-molecule antiviral drugs. At the population level, Alpha through Omicron variants do not demonstrate consistent resistance to remdesivir, nirmatrelvir/ritonavir, or molnupiravir [188].

Because virus-replication systems require the targets of antiviral drugs (polymerase and protease enzymes) to maintain their functional structures evolutionarily, these targets face limited mutant tolerance pressures compared to spike proteins. Multiple studies and surveillance tests from 2023 proved that antiviral agents remained effective against XBB.1.5, BQ.1.1, BA.2.86, and JN.1 strains [188]. Researchers from the University of Tokyo investigated the potency of remdesivir, molnupiravir, and nirmatrelvir against BQ.1.1 and XBB clinical patient strains and observed an IC50 change of less than 2-fold relative to the initial virus [96]. Existing antivirals maintain their effectiveness against B.2.86 and JN.1 because these variants have not developed Mpro (protease) mutations, while their main changes occur in spike and accessory proteins [189]. Antiviral medications used extensively might create conditions that would select resistant virus variants. Nirmatrelvir/ritonavir resistance mutations have not increased in global surveillance reports according to a JAMA study published in 2023 [190]. Isolated cases (primarily immunocompromised patients under extended treatment or experiencing a rebound) present Mpro E166V or L50F class mutations that make nirmatrelvir treatment ineffective [191]. Circulating Omicron viruses have not yet developed this complex mutation pattern in nature. The recent approval of the antiviral medication ensitrelvir gives medical teams multiple alternative treatments in case drug resistance occurs for currently available antivirals [105]. A new treatment strategy under consideration to prevent drug resistance involves the administration of two antivirals with different target mechanisms in combination therapy, like HIV or HCV treatment approaches. Clinical trials of combination therapy (nirmatrelvir/ritonavir + molnupiravir) are planned to test if a highly pathogenic drug-resistant variant appears [125].

For hospitalized patients requiring oxygen, dexamethasone is a recommended treatment. In cases of severe cytokine storm, administering tocilizumab or baricitinib is recommended. In such cases, administering high-flow nasal oxygen, non-invasive ventilation, or mechanical ventilation in intensive care unit settings is recommended [185]. Vaccination and boosters are crucial for reducing severe disease and hospitalization. Updated bivalent vaccines provide better protection against Omicron subvariants [187].

The natural infectivity of Omicron subvariants exhibits different levels of severity. The Omicron variant, starting with BA.1 (and later subvariants), produced milder respiratory tract infections than Delta because it showed different tissue cell preferences, targeting bronchi rather than lung alveoli. Omicron wave deaths declined due to population-based immunity, along with what might be considered a reduced disease severity, during XBB.1.5 and BQ.1.1 waves. The JN.1 coronavirus variant reduced hospital admission risks in U.S. patients by 40 percent, since its odds ratio was found to be approximately 0.60 compared to EG.5 Eris patients in late 2023 by American researchers [192]. Research found that the subvariant HV.1 presented a much lower hospitalization risk (a factor of 0.35 versus EG.5) than its descendant from EG.5. The disease severity decreased even more after Omicron sublineages progressed from EG.5 to HV.1 to JN.1 during late 2023, according to observational data [192]. Under immunological pressure and wide population immunity, the virus shows a preference for more transmissible strains while demonstrating less virulent characteristics. However, the average severity reduction does not ensure mild illness throughout all groups. Hospital admissions of high-risk populations increased during each Omicron wave because the number of infections increased. The highly immune-evasive variants BA.2.86 and JN.1 can infect people who are not recently boosted or exposed, including medically vulnerable individuals, which can result in pneumonia [193]. Treatments continue to be in high demand due to persistent patient needs. Investigations show that Omicron subvariants failed to gain resistance against pneumonia medication, including dexamethasone, tocilizumab, and other anti-inflammatory treatments [194]. These treatments function by modulating human immune responses; thus, variant-specific changes do not affect their therapeutic value. The milder virulent nature of Omicron leads to decreased patient progression toward the hyperinflammatory stage, at which point medical drugs are necessary. These treatments have led to better patient outcomes among the infected population. Hospital deaths among COVID-19 pneumonia patients declined markedly from 2020 to 2023 because Omicron caused less severe disease, while medical treatments (steroids and immunomodulators) and antiviral medication were utilized early on [195]. Absolute risk reductions from antiviral medications have decreased in communities where booster doses and previous infections are common, mainly because the basic illness severity is lower among these populations [196]. Healthcare providers should prescribe antiviral treatment to vulnerable patients, such as those over 75 years old and those with multiple comorbidities or immunosuppression issues, to prevent pneumonia from becoming life-threatening [197]. During the Omicron era, healthcare activities continue to focus on providing high-risk populations with prompt antiviral treatments to prevent hospitalization and enhance pneumonia care for those who need it.

## 5. Prognosis and Outcome of Severe COVID-19 Pneumonia

The outcomes of severe pneumonia caused by SARS-CoV-2 can vary widely depending on factors such as the patient’s age, underlying health conditions, timely medical intervention, and the severity of the disease. Severe pneumonia associated with COVID-19 is a significant contributor to morbidity and mortality, especially in patients with risk factors such as older age, obesity, cardiovascular disease, diabetes, and immunosuppressive conditions [197]. The recovery of patients who develop severe COVID-19 pneumonia takes an extended amount of time. In the process of recovery, hospitalization and rehabilitation, together with post-ICU care, typically take between weeks and months [198]. A subset of patients exhibits a positive recovery outcome for the novel strain of coronaviruses, while a significant proportion of cases result in protracted sequelae. The mortality rate associated with severe cases of pneumonia due to the novel coronavirus is particularly high among elderly patients and those with pre-existing health conditions. The mortality statistics for patients requiring mechanical ventilation due to severe pneumonia or experiencing ARDS is 30–50% or higher, based on the patient demographics examined [199]. Varying research studies have demonstrated ICU mortality statistics between 30% and 60% for patients with severe COVID-19 pneumonia who need mechanical ventilation or ECMO support, as their treatment course improved following better treatment protocols and vaccination strategies [200]. Acute kidney injury (AKI) in patients with COVID-19 pneumonia has been correlated with the need for ICU hospitalization, elevated inflammatory markers, and higher mortality. Chronic kidney disease (CKD) also causes higher mortality in patients with COVID-19 [201]. Patients receiving extended ICU care, along with those requiring mechanical ventilation, often show delayed recovery even after discharge from the hospital. The persistent medical impacts of COVID-19, known as Long COVID-19, consist of general fatigue along with mental processing difficulties known as “brain fog,” while patients can also experience muscle dysfunction and unusual breathing symptoms, along with joint discomfort, chest pressure, and difficulty breathing [202]. Mild COVID-19 infections have also been reported to lead to Long COVID, and those who suffered from severe pneumonia are at an increased risk of the condition.

Patients who require extended ICU care with ventilator support frequently develop Post-Intensive Care Syndrome (PICS) because extended ICU stays result in physical, cognitive, and psychological disabilities [203]. Medical complications affect patients by causing muscle wasting, anxiety, and depression, along with memory dysfunction, so they experience long-term effects on their recovery, along with a poor quality of life.

The various health conditions determine both the patient’s outcome and the progression of the disease in severe COVID-19 pneumonia. Older adults who reach the age of 65 and beyond face heightened risks of prolonged hospital stays and mortality [204]. Severe pneumonia leads to worse patient outcomes for those who have pre-existing medical conditions, including diabetes, hypertension, cardiovascular diseases, chronic respiratory diseases (e.g., COPD, asthma), and obesity [205]. Individuals with a diminished immune function stemming from cancer treatments or organ transplants and autoimmune diseases, or immunosuppressive medications have a heightened risk of severe pneumonia and infections due to their weakened defense mechanisms against the virus [206]. Research findings indicate that men face a marginally greater possibility of developing serious pneumonia and dying from it than women, although the risk differences are not absolute [207]. Some studies have shown that physical activity is correlated with a decreased need for hospitalization in patients diagnosed with COVID-19, reduced ICU admissions, and decreased mortality. Also, COVID-19 patients with a 908 history of resistance and endurance exercise have a lower rate of hospitalization and lower mortality [208].

Detailed antiviral treatments, together with corticosteroid therapy (dexamethasone), supplemental oxygen support, and additional care mechanisms, help patients recover effectively [209]. Delayed care, especially in patients who develop ARDS or other complications, can lead to worsened outcomes. The delivery of healthcare services later than needed can result in patients developing ARDS or other complications, which ultimately causes their condition to deteriorate. ARDS stems from severe pneumonia, with its most severe consequences being widespread lung inflammation and extensive fluid accumulation. The disease is recognized as fatal. ARDS patients need to receive mechanical ventilation therapy because their condition results in elevated death rates [210].

Hospitalized patients and ventilator-assisted patients experience an increased risk of both bacterial and fungal complications of pneumonia that augment mortality rates. Overall, ventilator-associated pneumonia is the most widespread infection among patients hospitalized in the ICU [211]. The extreme manifestation of COVID-19 pneumonia creates blood-clotting conditions that potentially lead to pulmonary embolism, DVT, and other thromboembolic complications [212]. Coronavirus disease causes damage to heart tissue, resulting in myocarditis or arrhythmias, besides injury to kidneys, which may worsen both the treatment course and disease outcome [213]. Research indicates that people who have received the full vaccination course experience minimized SARS-CoV-2 pneumonia severity. Extant research demonstrates that individuals who have been vaccinated are less likely to require hospital treatment and face a reduced risk of admission to intensive care units and death [214]. The chances of invasive mechanical ventilation are decreased among vaccinated patients, and such patients generally face milder disease severity when they develop COVID-19 infections. New forms of SARS-CoV-2 drive vaccine changes to boost performance, which, along with continuous booster doses, help limit severe cases.

Childhood severe pneumonia rarely appears compared to adult pneumonia cases and leads to better treatment results when patients develop it [214]. However, the post-viral complication known as multisystem inflammatory syndrome in children (MIS-C) is a consequence of childhood COVID-19 infection [215]. Pregnant women may develop severe pneumonia along with COVID-19 complications. The COVID-19 hyperinflammatory state induces a poor clinical result in pregnant women with severe pneumonia, who face an increased danger of premature birth combined with fetal distress and other medical conditions [216]. Survivors of severe pneumonia and ARDS require rehabilitation after their illness to restore their muscles, along with their lungs and physical abilities. Medical care through pulmonary rehabilitation facilities providing exercise training and instruction leads to substantial improvements for these patients [217]. Supportive clinical care through scheduled follow-up visits enables the tracking of lung health and post-intensive care syndrome management, along with PTSD and anxiety treatment, to achieve complete recovery.

## 6. Future Outlook

In 2023, the requirement for continuous development and transformation of our medicine supplies was highlighted. We can expect updated versions of existing drugs to be developed—for example, improved formulations of remdesivir or pan-coronavirus antivirals. Scientists are currently investigating a wide range of antiviral medications that target host elements such as TMPRSS2 inhibitors and drugs that control proteases from various coronavirus species to develop protection strategies for future coronavirus outbreaks, according to research presented in [218]. A specific compound functions as an inhibitor for the SARS-CoV-2 entry host enzyme and demonstrates potential effectiveness against any COVID-19 variant. Safe and tolerable host-targeted antiviral drugs avoid resistance yet remain effective. Researchers are also exploring inhaled therapy options, which allow drugs to directly reach the respiratory tract in order to achieve optimal lung drug concentration levels. A large trial in 2022 confirmed the findings of earlier trials but failed to demonstrate primary effectiveness when testing inhaled interferon beta (SNG001) for treating COVID-19. The administration of such therapies for selected patient groups or as combined treatment modalities may provide therapeutic value. The prevention of severe disease primarily relies on vaccines, though booster formulations now need to be updated based on XBB.1.5 variant changes. The safety net on which treatments function stands as protection against infections. The relationship between vaccines and treatments is important because appropriate booster shots reduce the number of patients needing treatment, and effective treatments ensure hospitals are not at capacity during vaccine-resistant outbreaks [196].

## 7. Conclusions

This review demonstrated that the management of severe pneumonia due to SARS-CoV-2 is predicated on maintaining oxygenation, treating the viral infection, controlling inflammation, and addressing complications. Treating severe pneumonia due to SARS-CoV-2 necessitates the expertise of multiple medical specialists, the administration of antiviral agents alongside corticosteroids and immunomodulatory therapies, the provision of ventilatory support, the administration of anticoagulants, and the utilization of other available therapies. The severity of illness determines the necessity of sophisticated treatment options, including mechanical ventilation or ECMO. Combining rapid medical diagnosis and patient-tailored treatment methods is critical to achieving optimal therapeutic outcomes for these patients. Moreover, the therapeutic strategy is adapted based on the patient’s current clinical state, existing medical conditions, and the severity of the underlying disease, and the outcomes of severe pneumonia due to SARS-CoV-2 can range from full recovery to long-term disability or death. These outcomes are influenced by various patient characteristics, including age, comorbidities, proper medical intervention, and vaccination status. While patients who receive prompt medical care generally recover, persistent disease symptoms from Long COVID and post-ICU syndrome can compromise their quality of life, necessitating ongoing medical attention. The advent of vaccines, in conjunction with the evolution of treatment modalities, has led to a marked improvement in the long-term prognoses for patients afflicted with severe pneumonia due to SARS-CoV-2. Nevertheless, specific challenges persist, particularly among vulnerable risk groups. Investment in pan-coronavirus drugs and universal coronavirus vaccines could eventually make variant-specific issues less acute. For now, the strategy is vigilance and agility, detecting new variants early, assessing their sensitivity to existing treatments, and deploying updated therapeutics as needed. In 2025, it is clear that while SARS-CoV-2 continues to evolve, the medical community has likewise evolved its armamentarium.

## Figures and Tables

**Table 1:** Ventilatory Support Strategies for COVID-19 Patients [69,77,78,79,80,81].

Stage	Criteria	Ventilatory Strategy	Key Considerations
Early Stage (Mild to Moderate Hypoxemia)	SpO_2_ 90–96% on nasal cannula; RR < 30	Nasal Cannula (2–6 L/min); High-Flow Nasal Oxygen (HFNO) (30–60 L/min, FiO_2_ titrated)	Monitor for increasing oxygen demand. FNO preferred over NIV due to better tolerance.
Progressive Hypoxemia (Failure of HFNO/NIV)	SpO_2_ < 90% despite HFNO; RR > 30; signs of fatigue	Non-Invasive Ventilation (NIV, CPAP/BiPAP) (CPAP 5–10 cmH_2_O, or BiPAP: IPAP 10–15 cmH_2_O, EPAP 5–10 cmH_2_O)	Aerosol-generating; use in negative pressure room if possible. If worsening, proceed to intubation.
Moderate to Severe ARDS (Intubation Required)	SpO_2_ < 88%, RR > 30, PaO_2_/FiO_2_ < 200	Lung-Protective Ventilation (VT = 6 mL/kg PBW, PEEP 10–15 cmH_2_O); Prone Positioning (12–16 h/day if PaO_2_/FiO_2_ < 150); Neuromuscular Blockade if dyssynchrony	Avoid high tidal volumes to prevent ventilator-induced lung injury (VILI). Conservative fluid management.
Severe ARDS/Refractory Hypoxemia	PaO_2_/FiO_2_ < 80 despite ventilation	Rescue Therapies: - Higher PEEP (>15 cm H_2_O); Inhaled Pulmonary Vasodilators (NO, epoprostenol); Neuromuscular blockade (48 h)	Careful PEEP titration to avoid overdistension. - High risk of barotrauma.
ECMO Consideration (Last Resort)	PaO_2_/FiO_2_ < 80, pH < 7.15, high plateau pressures (>35 cmH_2_O)	VV-ECMO (Venovenous ECMO)	Age < 65, no multi-organ failure.
Weaning from Ventilation	SpO_2_ > 94% on FiO_2_ < 40%, PEEP ≤ 8	Spontaneous Breathing Trial (SBT) (PSV trial); HFNO or NIV post-extubation	Swallow assessment before oral intake to prevent aspiration.

**Table 2:** Key Antiviral and Pharmacologic Treatments for COVID-19.

Treatment (Route)	Mechanism of Action	Key Efficacy Data (Clinical Trials)	Regulatory Status (as of 2025)	Activity vs. Omicron Variants
Nirmatrelvir + Ritonavir (Paxlovid, oral)	3CL protease inhibitor (blocks viral replication), boosted by ritonavir for PK.	89% reduction in hospitalization or death in high-risk unvaccinated (EPIC-HR trial) [121]. Observational studies show benefit in vaccinated patients, though absolute risk reduction is smaller.	FDA Approved (2023) for high-risk adults [128]; EUA for ≥12 year pediatric. Widely recommended first-line outpatient therapy.	Active against all Omicron subvariants. Targets conserved protease, so efficacy is unaffected by spike mutations [122]. No widespread resistance observed [123,124].
Remdesivir (Veklury, IV)	Nucleoside analog; inhibits RNA-dependent RNA polymerase (viral replication).	87% reduction in hospitalization (3-day outpatient course) shortened recovery time by ~5 days in hospitalized (ACTT-1). May improve survival when given early.	FDA Approved (2020) for hospitalized adults; expanded to severe renal impairment (2022) [129]. Standard care for moderate-to-severe hospitalized COVID.	Active against all variants. Polymerase target conserved; retains potency vs. XBB.1.5, BQ.1.1, BA.2.86, etc. [114,115]. Rare resistance mutations in immunocompromised patients, usually with fitness cost [88,89].
Molnupiravir (Lagevrio, oral)	Nucleoside analog; induces lethal mutagenesis in viral RNA.	~30% reduction in hospitalization in high-risk unvaccinated (MOVe-OUT trial). No significant effect on mortality. Lower efficacy than other antivirals.	FDA EUA (2021–2025) for high-risk adults only if other options are unavailable [128]. Not first-line; use declining.	Active against all variants (targets polymerase). However, induces SARS-CoV-2 mutations; molnupiravir-linked mutation signatures detected in global sequences [129,130]. No evidence of more pathogenic variants from this, but use sparingly to avoid resistance.
Ensitrelvir (Xocova, oral)	3CL protease inhibitor (once-daily dosing; no ritonavir required).	Shortened duration of COVID-19 symptoms by ~24 h in largely vaccinated, low-risk. patients (Phase 3 SCORPIO-SR) [131,132]. Met the primary endpoint for faster symptom resolution (*p* = 0.04). Demonstrated viral load reduction vs. placebo.	Approved in Japan (2022–2023) [133]; Singapore (2023). Not yet approved in the U.S./EU (Fast Track status). Phase 3 trials are ongoing globally.	Presumed active vs. Omicron subvariants (same target as nirmatrelvir). Tested during BA.2 wave with success [134]. In vitro studies show no resistance issues to date. Monitoring ongoing for emergent protease mutations.
Peginterferon Lambda (SC injection)	Pegylated type III interferon; stimulates antiviral immunity in lung epithelium (host-targeted).	~50% reduction in hospitalization/emergency visits in outpatients (TOGETHER trial, mixed variants) [130]. Benefits observed even in vaccinated patients. Single dose administration.	Not approved (investigational). Positive Phase 3 published in 2023; FDA declined EUA due to trial scope. May see further development for immunocompromised host therapy.	Active against all variants (boosts host immune response)—efficacy is variant-agnostic [130]. Would not be affected by viral spike mutations or antiviral resistance mechanisms.
Anakinra (Kineret, IV)	Interleukin-1 receptor antagonist; immunomodulator reducing inflammation.	In hospitalized COVID pneumonia patients with high inflammation (elevated suPAR), anakinra improved outcomes (SAVE-MORE trial earlier). Reduction in progression to respiratory failure reported.	FDA EUA (November 2022) for COVID pneumonia requiring oxygen in adults with high suPAR (immune activation marker) [128]. Approved in EU for COVID with cytokine release risk.	Variant-independent (targets host). Efficacy linked to hyperinflammatory state rather than viral characteristics.
Baricitinib (Olumiant, oral)	Janus kinase (JAK1/JAK2) inhibitor; dampens cytokine signaling (e.g., IL-6, interferons).	~5% absolute reduction in 28-day mortality in hospitalized patients on oxygen (ACTT-2, COV-BARRIER trials). Accelerated recovery when added to standard care [128]. Benefit seen on top of steroids.	FDA Approved (May 2022) for hospitalized adults on oxygen/ventilator [128]. Incorporated into NIH/WHO treatment guidelines for severe COVID.	Variant-independent. Addresses downstream hyperinflammation. Remains effective regardless of viral variant (host-targeted immunomodulation).
Tocilizumab (Actemra, IV)	IL-6 receptor monoclonal antibody; mitigates cytokine storm and immunopathology.	4–8% reduction in mortality in severe COVID when given with steroids (RECOVERY, REMAP-CAP trials) [128]. In RECOVERY, mortality was 29% vs. 33% in controls; improved odds of discharge.	FDA Approved (December 2022) for hospitalized adults on systemic steroids and oxygen/ventilatory support [128]. WHO-recommended for severe/critical cases.	Variant-independent. Moderates host immune response. Effectiveness not affected by changes in viral spike proteins.
Sabizabulin (oral)	Microtubule inhibitor; dual antiviral and anti-inflammatory actions (disrupts viral transport and dampens lung inflammation).	In a Phase 3 trial of critically ill COVID patients, sabizabulin reduced 60-day mortality from 45% (placebo) to 20% (sabizabulin) [108]. A 55% relative risk reduction. Small sample (150 patients) but marked benefit.	Not approved; FDA Fast Track status. EUA request (2022) was denied pending a larger confirmatory trial [134]. A new global Phase 3 trial (≈408 patients) is underway as of 2023 [134].	Variant-independent (targets host cell processes). Would be expected to retain efficacy across variants. Needs confirmation in broader studies before clinical adoption.

Abbreviations: PK = pharmacokinetics; EUA = Emergency Use Authorization; suPAR = soluble urokinase plasminogen activator receptor.

**Table 3:** The pharmacologic treatment for severe and critically ill COVID-19 patients.

Severity	Recommended Treatments	Not Recommended
Severe COVID-19	Corticosteroids (Dexamethasone preferred) Remdesivir (may decrease recovery time but no mortality benefit) IL-6 Inhibitors (Tocilizumab, Sarilumab) JAK Inhibitors (Baricitinib preferred over Tofacitinib)	Hydroxychloroquine Azithromycin Lopinavir/Ritonavir Convalescent plasma Ivermectin (outside clinical trials)
Critically Ill (Non-Invasive Ventilation/HFNC)	Corticosteroids (Dexamethasone preferred, Hydrocortisone alternative) IL-6 Inhibitors (Tocilizumab preferred over Sarilumab) JAK Inhibitors (Baricitinib preferred over Tofacitinib)	Remdesivir (no benefit in this population)
Critically Ill (Invasive Mechanical Ventilation/ECMO)	Corticosteroids (Dexamethasone preferred) IL-6 Inhibitors (Tocilizumab preferred over Sarilumab) Baricitinib (if IL-6 inhibitors not available)	Most other COVID-19 therapies lack evidence or have not been studied in this population

## Data Availability

Data sharing is not applicable.

## Abbreviations

The following abbreviations are used in this manuscript:

AC	Assist-Control
ACE2	Angiotensin-converting enzyme 2 receptors
aPTT	Activated Partial Thromboplastin Time
ARDS	Acute respiratory distress syndrome
ABG	Arterial Blood Gas
BiPAP	Bilevel positive airway pressure
CPAC	Continuous positive airway pressure
CRP	C- reactive protein
CT	Computer tomography
DIC	Disseminated intravascular coagulation
DVT	Deep vein thrombosis
DOAC	Direct oral anticoagulants
ECG	Electrocardiogram
ECMO	Extracorporeal membrane oxygenation
HFNC	High-Flow Nasal Cannula
HRCT	High-Resolution Chest CT Scan
IL	Interleukins
INR	International Normalized Ratio
LMWH	Low-molecular-weight heparin
MIS-C	Multisystem inflammatory syndrome in children
NIC	Non-invasive ventilation
PCR	Polymerase chain reaction
PE	Pulmonary embolism
PEEP	Positive End-Expiratory Pressure
PICS	Post-Intensive Care Syndrome
PT	Prothrombin time
RT-PCR	Reverse transcription polymerase chain reaction
SBT	Spontaneous Breathing Trials
TNF-α	Tumor Necrosis Factor
VILI	Ventilator-induced lung injury
VTE	Venous thromboembolism

## References

[1] Wu Z., McGoogan J.M. (2020). Characteristics of and Important Lessons From the Coronavirus Disease 2019 (COVID-19) Outbreak in China. JAMA J. Am. Med. Assoc..

[2] Hu B., Guo H., Zhou P., Shi Z.-L. (2021). Characteristics of SARS-CoV-2 and COVID-19. Nat. Rev. Microbiol..

[3] Grasselli G., Zangrillo A., Zanella A., Antonelli M., Cabrini L., Castelli A., Cereda D., Coluccello A., Foti G., Fumagalli R. (2020). Baseline Characteristics and Outcomes of 1591 Patients Infected With SARS-CoV-2 Admitted to ICUs of the Lombardy Region, Italy. JAMA.

[4] Lamers M.M., Haagmans B.L. (2022). SARS-CoV-2 pathogenesis. Nat. Rev. Microbiol..

[5] Iwasaki M., Saito J., Zhao H., Sakamoto A., Hirota K., Ma D. (2021). Inflammation triggered by SARS-CoV-2 and ACE2 augment drives multiple organ failure of severe COVID-19: Molecular mechanisms and implications. Inflammation.

[6] Zhou P., Yang X.L., Wang X.G., Hu B., Zhang L., Zhang W., Si H.-R., Zhu Y., Li B., Huang C.-L. (2020). A pneumonia outbreak associated with a new coronavirus of probable bat origin. Nature.

[7] Lan J., Ge J., Yu J., Shan S., Zhou H., Fan S., Zhang Q., Shi X., Wang Q., Zhang L. (2020). Structure of the SARS-CoV-2 spike receptor-binding domain bound to the ACE2 receptor. Nature.

[8] Hoffmann M., Kleine-Weber H., Schroeder S., Krüger N., Herrler T., Erichsen S., Schiergens T.S., Herrler G., Wu N.-H., Nitsche A. (2020). SARS-CoV-2 cell entry depends on ACE2 and TMPRSS2 and is blocked by a clinically proven protease inhibitor. Cell.

[9] Walls A.C., Park Y.-J., Tortorici M.A., Wall A., McGuire A.T., Veesler D. (2020). Structure, Function, and Antigenicity of the SARS-CoV-2 Spike Glycoprotein. Cell.

[10] Yin X., Riva L., Pu Y., Martin-Sancho L., Kanamune J., Yamamoto Y., Sakai K., Gotoh S., Miorin L., De Jesus P.D. (2021). MDA5 Governs the Innate Immune Response to SARS-CoV-2 in Lung Epithelial Cells. Cell Rep..

[11] Khanmohammadi S., Rezaei N. (2021). Role of Toll-like receptors in the pathogenesis of COVID-19. J. Med. Virol..

[12] Vora S.M., Lieberman J., Wu H. (2021). Inflammasome activation at the crux of severe COVID-19. Nat. Rev. Immunol..

[13] Siu Y.L., Teoh K.T., Lo J., Chan C.M., Kien F., Escriou N., Tsao S.W., Nicholls J.M., Altmeyer R., Peiris J.S.M. (2008). The M, E, and N Structural Proteins of the Severe Acute Respiratory Syndrome Coronavirus Are Required for Efficient Assembly, Trafficking, and Release of Virus-Like Particles. J. Virol..

[14] Hadjadj J., Yatim N., Barnabei L., Corneau A., Boussier J., Smith N., Péré H., Charbit B., Bondet V., Chenevier-Gobeaux C. (2020). Impaired type I interferon activity and inflammatory responses in severe COVID-19 patients. Science.

[15] Ferreira A.C., Soares V.C., de Azevedo-Quintanilha I.G., Dias S.d.S.G., Fintelman-Rodrigues N., Sacramento C.Q., Mattos M., de Freitas C.S., Temerozo J.R., Teixeira L. (2021). SARS-CoV-2 engages inflammasome and pyroptosis in human primary monocytes. Cell Death Discov..

[16] Zhang J., Wu H., Yao X., Zhang D., Zhou Y., Fu B., Wang W., Li H., Wang Z., Hu Z. (2021). Pyroptotic macrophages stimulate the SARS-CoV-2-associated cytokine storm. Cell. Mol. Immunol..

[17] Chen R., Lan Z., Ye J., Pang L., Liu Y., Wu W., Qin X., Guo Y., Zhang P. (2021). Cytokine Storm: The Primary Determinant for the Pathophysiological Evolution of COVID-19 Deterioration. Front. Immunol..

[18] Karki R., Sharma B.R., Tuladhar S., Williams E.P., Zalduondo L., Samir P., Zheng M., Sundaram B., Banoth B., Malireddi R.K.S. (2021). Synergism of TNF-α and IFN-γ Triggers Inflammatory Cell Death, Tissue Damage, and Mortality in SARS-CoV-2 Infection and Cytokine Shock Syndromes. Cell.

[19] Liu Y., Yang Y., Zhang C., Huang F., Wang F., Yuan J., Wang Z., Li J., Li J., Feng C. (2020). Clinical and biochemical indexes from 2019-nCoV infected patients linked to viral loads and lung injury. Sci. China Life Sci..

[20] Cardinal-Fernández P., Lorente J.A., Ballén-Barragán A., Matute-Bello G. (2017). Acute respiratory distress syndrome and diffuse alveolar damage. new insights on a complex relationship. Ann. Am. Thorac. Soc..

[21] Carsana L., Sonzogni A., Nasr A., Rossi R.S., Pellegrinelli A., Zerbi P., Rech R., Colombo R., Antinori S., Corbellino M. (2020). Pulmonary post-mortem findings in a series of COVID-19 cases from northern Italy: A two-centre descriptive study. Lancet Infect. Dis..

[22] Ruan Q., Yang K., Wang W., Jiang L., Song J. (2020). Clinical predictors of mortality due to COVID-19 based on an analysis of data of 150 patients from Wuhan, China. Intensive Care Med..

[23] Menter T., Haslbauer J.D., Nienhold R., Savic S., Hopfer H., Deigendesch N., Frank S., Turek D., Willi N., Pargger H. (2020). Post-mortem examination of COVID-19 patients reveals diffuse alveolar damage with severe capillary congestion and variegated findings in lungs and other organs suggesting vascular dysfunction. Histopathology.

[24] Sinha A., Deshwal H., Vashisht R. (2025). Mechanical Ventilation and Extracorporeal Membrane Oxygenation Considerations in COVID-19. StatPearls.

[25] Delorey T.M., Ziegler C.G.K., Heimberg G., Normand R., Yang Y., Segerstolpe Å., Abbondanza D., Fleming S.J., Subramanian A., Montoro D.T. (2021). COVID-19 tissue atlases reveal SARS-CoV-2 pathology and cellular targets. Nature.

[26] Wichmann D., Sperhake J.P., Lütgehetmann M., Steurer S., Edler C., Heinemann A., Heinrich F., Mushumba H., Kniep I., Schröder A.S. (2020). Autopsy Findings and Venous Thromboembolism in Patients with COVID-19. Ann. Intern. Med..

[27] Xiang-Hua Y., Le-Min W., Ai-Bin L., Zhu G., Riquan L., Xu-You Z., Wei-Wei R., Ye-Nan W. (2010). Severe Acute Respiratory Syndrome and Venous Thromboembolism in Multiple Organs. Am. J. Respir. Crit. Care Med..

[28] Cunningham R.M.J., Moore K.L., Moore J.S. (2022). Coagulopathy during COVID-19 infection: A brief review. Clin. Exp. Med..

[29] Zhang Y., Xiao M., Zhang S., Xia P., Cao W., Jiang W., Chen H., Ding X., Zhao H., Zhang H. (2020). Coagulopathy and Antiphospholipid Antibodies in Patients with Covid-19. N. Engl. J. Med..

[30] Al-Samkari H., Leaf R.S.K., Dzik W.H., Carlson J.C.T., Fogerty A.E., Waheed A., Goodarzi K., Bendapudi P.K., Bornikova L., Gupta S. (2020). COVID-19 and coagulation: Bleeding and thrombotic manifestations of SARS-CoV-2 infection. Blood.

[31] Klok F.A., Kruip M.J.H.A., van der Meer N.J.M., Arbous M.S., Gommers D., Kant K.M., Kaptein F.H.J., van Paassen J., Stals M.A.M., Huisman M.V. (2020). Confirmation of the high cumulative incidence of thrombotic complications in critically ill ICU patients with COVID-19: An updated analysis. Thromb. Res..

[32] Grasselli G., Tonetti T., Protti A., Langer T., Girardis M., Bellani G., Laffey J., Carrafiello G., Carsana L., Rizzuto C. (2020). Pathophysiology of COVID-19-associated acute respiratory distress syndrome: A multicentre prospective observational study. Lancet Respir. Med..

[33] Bonaventura A., Vecchié A., Dagna L., Martinod K., Dixon D.L., Van Tassell B.W., Dentali F., Montecucco F., Massberg S., Levi M. (2021). Endothelial dysfunction and immunothrombosis as key pathogenic mechanisms in COVID-19. Nat. Rev. Immunol..

[34] Tang N., Li D., Wang X., Sun Z. (2020). Abnormal coagulation parameters are associated with poor prognosis in patients with novel coronavirus pneumonia. J. Thromb. Haemost..

[35] Rentsch C.T., Beckman J.A., Tomlinson L., Gellad W.F., Alcorn C., Kidwai-Khan F., Skanderson M., Brittain E., King J.T., Ho Y.-L. (2021). Early initiation of prophylactic anticoagulation for prevention of coronavirus disease 2019 mortality in patients admitted to hospital in the United States: Cohort study. BMJ.

[36] Naseef H.A., Mohammad U., Al-Shami N., Sahoury Y., Abukhalil A.D., Dreidi M., Alsahouri I., Farraj M. (2022). Bacterial and fungal co-infections among ICU COVID-19 hospitalized patients in a Palestinian hospital: A retrospective cross-sectional study. F1000Research.

[37] Rabaan A.A., Smajlović S., Tombuloglu H., Ćordić S., Hajdarević A., Kudić N., Al Mutai A., Turkistani S.A., Al-Ahmed S.H., Al-Zaki N.A. (2022). SARS-CoV-2 infection and multi-organ system damage: A review. Bosn. J. Basic Med. Sci..

[38] Peluso M.J., Deeks S.G. (2024). Mechanisms of long COVID and the path toward therapeutics. Cell.

[39] Khaswal A., Kumar V., Kumar S. (2022). Long-Term Health Consequences of SARS-CoV-2: Assumptions Based on SARS-CoV-1 and MERS-CoV Infections. Diagnostics.

[40] Chodick G. (2025). Long-term infectious sequelae after SARS-CoV-2 infection should be considered in mild cases too. Lancet Infect. Dis..

[41] Ewing A.G., Salamon S., Pretorius E., Joffe D., Fox G., Bilodeau S., Bar-Yam Y. (2024). Review of organ damage from COVID and Long COVID: A disease with a spectrum of pathology. Med. Rev..

[42] Gheorghita R., Soldanescu I., Lobiuc A., Sturdza O.A.C., Filip R., Constantinescu–Bercu A., Dimian M., Mangul S., Covasa M. (2024). The knowns and unknowns of long COVID-19: From mechanisms to therapeutical approaches. Front. Immunol..

[43] Sun Z., Shi C., Jin L. (2024). Mechanisms by Which SARS-CoV-2 Invades and Damages the Central Nervous System: Apart from the Immune Response and Inflammatory Storm, What Else Do We Know?. Viruses.

[44] Caliman-Sturdza O.A., Gheorghita R., Lobiuc A. (2025). Neuropsychiatric Manifestations of Long COVID-19: A Narrative Review of Clinical Aspects and Therapeutic Approaches. Life.

[45] Naughton S.X., Raval U., Pasinetti G.M. (2020). Potential Novel Role of COVID-19 in Alzheimer’s Disease and Preventative Mitigation Strategies. J. Alzheimer’s Dis..

[46] Satterfield B.A., Bhatt D.L., Gersh B.J. (2022). Cardiac involvement in the long-term implications of COVID-19. Nat. Rev. Cardiol..

[47] Xie Y., Xu E., Bowe B., Al-Aly Z. (2022). Long-term cardiovascular outcomes of COVID-19. Nat. Med..

[48] Huang C., Wang Y., Li X., Ren L., Zhao J., Hu Y., Zhang L., Fan G., Xu J., Gu X. (2020). Clinical features of patients infected with 2019 novel coronavirus in Wuhan, China. Lancet.

[49] Attaway A.H., Scheraga R.G., Bhimraj A., Biehl M., Hatipoğlu U. (2021). Severe COVID-19 pneumonia: Pathogenesis and clinical management. BMJ.

[50] Goyal D., Inada-Kim M., Mansab F., Iqbal A., McKinstry B., Naasan A.P., Millar C., Thomas S., Bhatti S., Lasserson D. (2021). Improving the early identification of COVID-19 pneumonia: A narrative review. BMJ Open Respir. Res..

[51] Shao C., Shi Y., Chen R., Liu X., Huang H., Zhao Y., Xu K., Chen K., Wang M., Xu Z. (2023). Risk factors associated with COVID-19 pneumonia in Chinese patients with pre-existing interstitial lung disease during the SARS-CoV-2 pandemic. J. Med. Virol..

[52] Stokes E.K., Zambrano L.D., Anderson K.N., Marder E.P., Raz K.M., El Burai Felix S., Tie Y., Fullerton K.E. (2020). Coronavirus Disease 2019 Case Surveillance—United States, 22 January–30 May 2020. MMWR Morb. Mortal. Wkly. Rep..

[53] Hidalgo D.C., Olusanya O., Harlan E. (2021). Critical care trainees call for pulse oximetry reform. Lancet Respir. Med..

[54] Sanghani H., Bansal S., Parmar V., Shah R. (2022). Study of Arterial Blood Gas Analysis in Moderate-to-Severe COVID-19 Patients. Cureus.

[55] Wilson-Baig N. (2021). Happy hypoxia in COVID19: Pathophysiology and pulse oximetry accuracy. J. Paramed. Pract..

[56] Kimura H. (2022). Happy hypoxia in COVID19 patients associated with hypoxic ventilatory depression. Acta Med. Nagasakiensia.

[57] Dhont S., Derom E., Van Braeckel E., Depuydt P., Lambrecht B.N. (2020). The pathophysiology of ‘happy’ hypoxemia in COVID-19. Respir. Res..

[58] Wong H.Y.F., Lam H.Y.S., Fong A.H.-T., Leung S.T., Chin T.W.-Y., Lo C.S.Y., Lui M.M.-S., Lee J.C.Y., Chiu K.W.-H., Chung T.W.-H. (2020). Frequency and Distribution of Chest Radiographic Findings in Patients Positive for COVID-19. Radiology.

[59] Bao C., Liu X., Zhang H., Li Y., Liu J. (2020). Coronavirus Disease 2019 (COVID-19) CT Findings: A Systematic Review and Meta-analysis. J. Am. Coll. Radiol..

[60] Kwee T.C., Kwee R.M. (2020). Chest CT in COVID-19: What the Radiologist Needs to Know. RadioGraphics.

[61] Chung M., Bernheim A., Mei X., Zhang N., Huang M., Zeng X., Cui J., Xu W., Yang Y., Fayad Z.A. (2020). CT Imaging Features of 2019 Novel Coronavirus (2019–nCoV). Radiology.

[62] Rajpurkar P., Irvin J., Zhu K., Yang B., Mehta H., Duan T., Ding D., Bagul A., Ball R.L., Langlotz C. (2017). CheXNet: Radiologist-Level Pneumonia Detection on Chest X-Rays with Deep Learning. arXiv.

[63] Archana K., Kaur A., Gulzar Y., Hamid Y., Mir M.S., Soomro A.B. (2023). Deep learning models/techniques for COVID-19 detection: A survey. Front. Appl. Math. Stat..

[64] Ye Q., Gao Y., Ding W., Niu Z., Wang C., Jiang Y., Wang M., Fang E.F., Menpes-Smith W., Xia J. (2022). Robust weakly supervised learning for COVID-19 recognition using multi-center CT images. Appl. Soft Comput..

[65] Vaid S., Kalantar R., Bhandari M. (2020). Deep learning COVID-19 detection bias: Accuracy through artificial intelligence. Int. Orthop..

[66] Chowdhury M.E.H., Rahman T., Khandakar A., Mazhar R., Kadir M.A., Bin Mahbub Z., Islam K.R., Khan M.S., Iqbal A., Al Emadi N. (2020). Can AI Help in Screening Viral and COVID-19 Pneumonia?. IEEE Access.

[67] Hardy-Werbin M., Maiques J.M., Busto M., Cirera I., Aguirre A., Garcia-Gisbert N., Zuccarino F., Carbullanca S., Del Carpio L.A., Ramal D. (2023). MultiCOVID: A multi modal deep learning approach for COVID-19 diagnosis. Sci. Rep..

[68] Wiersinga W.J., Rhodes A., Cheng A.C., Peacock S.J., Prescott H.C. (2020). Pathophysiology, Transmission, Diagnosis, and Treatment of Coronavirus Disease 2019 (COVID-19). JAMA.

[69] Wang L., Lu S., Guo Y., Liu J., Wu P., Yang S. (2023). Comparative study of diagnostic efficacy of sputum and bronchoalveolar lavage fluid specimens in community-acquired pneumonia children treated with fiberoptic bronchoscopy. BMC Infect. Dis..

[70] Tomassetti S., Ciani L., Luzzi V., Gori L., Trigiani M., Giuntoli L., Lavorini F., Poletti V., Ravaglia C., Torrego A. (2024). Utility of bronchoalveolar lavage for COVID-19: A perspective from the Dragon consortium. Front. Med..

[71] Tong X., Cheng A., Yuan X., Zhong X., Wang H., Zhou W., Xu X., Li Y. (2021). Characteristics of peripheral white blood cells in COVID-19 patients revealed by a retrospective cohort study. BMC Infect. Dis..

[72] Ansari-Moghaddam B., Ahmadi S.A.Y., Matouri M., Ghaemmaghami A., Amiri A., Tavakkol E., Shahsavar F. (2022). Screening role of complete blood cell count indices and C reactive protein in patients who are symptomatic for COVID-19. Disaster Emerg. Med. J..

[73] Potempa L.A., Rajab I.M., Hart P.C., Bordon J., Fernandez-Botran R. (2020). Insights into the Use of C-Reactive Protein as a Diagnostic Index of Disease Severity in COVID-19 Infections. Am. J. Trop. Med. Hyg..

[74] Kurian S.J., Mathews S.P., Paul A., Viswam S.K., Nagri S.K., Miraj S.S., Karanth S. (2023). Association of serum ferritin with severity and clinical outcome in COVID-19 patients: An observational study in a tertiary healthcare facility. Clin. Epidemiol. Glob. Health.

[75] Hu R., Han C., Pei S., Yin M., Chen X. (2020). Procalcitonin levels in COVID-19 patients. Int. J. Antimicrob. Agents.

[76] Ibañez C., Perdomo J., Calvo A., Ferrando C., Reverter J.C., Tassies D., Blasi A. (2021). High D dimers and low global fibrinolysis coexist in COVID19 patients: What is going on in there?. J. Thromb. Thrombolysis.

[77] Wu H.-Y., Chang P.-H., Huang Y.-S., Tsai C.-S., Chen K.-Y., Lin I.-F., Hsih W.-H., Tsai W.-L., Chen J.-A., Yang T.-L. (2023). Recommendations and guidelines for the diagnosis and management of Coronavirus Disease-19 (COVID-19) associated bacterial and fungal infections in Taiwan. J. Microbiol. Immunol. Infect..

[78] Sagnelli C., Benito C., Monari C., Cirillo S., De Angelis G., Bianco A., Coppola N. (2020). Management of SARS-CoV-2 pneumonia. J. Med. Virol..

[79] Thirkell P., Griffiths M., Waller M.D. (2022). Management of Coronavirus Disease 2019 (COVID-19) Pneumonia. Encyclopedia of Respiratory Medicine.

[80] Cajanding R. (2022). Oxygen use and saturation targets in patients with COVID-19: Are we giving too much or aiming too low?. Nurs. Crit. Care.

[81] Fan E., Beitler J.R., Brochard L., Calfee C.S., Ferguson N.D., Slutsky A.S., Brodie D. (2020). COVID-19-associated acute respiratory distress syndrome: Is a different approach to management warranted?. Lancet Respir. Med..

[82] Ramadori G.P. (2022). SARS-CoV-2-Infection (COVID-19): Clinical Course, Viral Acute Respiratory Distress Syndrome (ARDS) and Cause(s) of Death. Med. Sci..

[83] Dondorp A.M., Hayat M., Aryal D., Beane A., Schultz M.J. (2020). Respiratory Support in COVID-19 Patients, with a Focus on Resource-Limited Settings. Am. J. Trop. Med. Hyg..

[84] Saffarini L., Sabobeh N., Lasfer C., Kazim S. (2024). High-Flow Nasal Cannula in COVID-19 Patients with Moderate to Severe Respiratory Distress: A Retrospective Analysis. Cureus.

[85] Dobler C.C., Murad M.H., Wilson M.E. (2020). Noninvasive Positive Pressure Ventilation in Patients With COVID-19. Mayo Clin. Proc..

[86] Groff P., Ferrari R. (2021). Non-invasive respiratory support in the treatment of acute hypoxemic respiratory failure secondary to CoViD-19 related pneumonia. Eur. J. Intern. Med..

[87] Tarif A.S., Salem M.B., Abdalrazik F.S. (2024). Clinical outcome of severe and critical covid patient receiving NIV or invasive ventilation. Egypt. J. Bronchol..

[88] Yau C.E., Lee D.Y.X., Vasudevan A., Goh K.J., Wong E., Ho A.F.W., Lim D.Y.Z. (2023). Performance of the ROX index in predicting high flow nasal cannula failure in COVID-19 patients: A systematic review and meta-analysis. Crit. Care.

[89] Kenaan M., Hyzy R. (2019). Mechanical Ventilation and Advanced Respiratory Support in the Cardiac Intensive Care Unit. Cardiac Intensive Care.

[90] Tanios M., Wu T.T., Nguyen H., Smith L., Mahidhara R., Devlin J.W. (2024). Comparing the impact of targeting limited driving pressure to low tidal volume ventilation on mortality in mechanically ventilated adults with COVID-19 ARDS: An exploratory target trial emulation. BMJ Open Respir. Res..

[91] Mora Carpio A.L., Mora J.I. (2025). Assist-Control Ventilation. StatPearls.

[92] Nijbroek S.G.L.H., Hol L., Ivanov D., Schultz M.J., Paulus F., Neto A.S. (2022). Low tidal volume ventilation is associated with mortality in COVID-19 patients—Insights from the PRoVENT-COVID study. J. Crit. Care.

[93] Abou-Arab O., Haye G., Beyls C., Huette P., Roger P.-A., Guilbart M., Bernasinski M., Besserve P., Trojette F., Dupont H. (2021). Hypoxemia and prone position in mechanically ventilated COVID-19 patients: A prospective cohort study. Can. J. Anaesth..

[94] Klaiman T., Silvestri J.A., Srinivasan T., Szymanski S., Tran T., Oredeko F., Sjoding M.W., Fuchs B.D., Maillie S., Jablonski J. (2021). Improving Prone Positioning for Severe Acute Respiratory Distress Syndrome during the COVID-19 Pandemic. An Implementation-Mapping Approach. Ann. Am. Thorac. Soc..

[95] Huang D., Tian H., Song W., Wang J., Yao Z., Xiong L., Jiang C., Zhang A., Ke X. (2024). Effects of innovative modular prone positioning tools in patients with acute respiratory distress syndrome due to COVID-19 during awake prone position: A prospective randomized controlled trial. Eur. J. Med. Res..

[96] Ma X., Liang M., Ding M., Liu W., Ma H., Zhou X., Ren H. (2020). Extracorporeal Membrane Oxygenation (ECMO) in Critically Ill Patients with Coronavirus Disease 2019 (COVID-19) Pneumonia and Acute Respiratory Distress Syndrome (ARDS). Med. Sci. Monit..

[97] Alessandri F., Di Nardo M., Ramanathan K., Brodie D., MacLaren G. (2023). Extracorporeal membrane oxygenation for COVID-19-related acute respiratory distress syndrome: A narrative review. J. Intensive Care.

[98] Lee S.-I., Kang D.H., Ahn H.J., Kim M.J., Shim M.-S., Lee J.E. (2022). Age is an important prognostic factor in COVID-19 patients treated with extracorporeal membrane oxygenation. J. Thorac. Dis..

[99] Li X., Hu M., Zheng R., Wang Y., Kang H., Jiang L., Zhong M., Sang L., Zheng X., Pan C. (2021). Delayed Initiation of ECMO Is Associated With Poor Outcomes in Patients With Severe COVID-19: A Multicenter Retrospective Cohort Study. Front. Med..

[100] Lee S., Kang G., Song S., Lee K., Yoo W., Jang H., Jang M.H. (2024). Factors Associated with Outcomes of Patients with Veno-Venous Extracorporeal Membrane Oxygenation for COVID-19. J. Clin. Med..

[101] Rudym D., Pham T., Rackley C.R., Grasselli G., Anderson M., Baldwin M.R., Beitler J., Agerstrand C., Serra A., Winston L.A. (2023). Mortality in Patients with Obesity and Acute Respiratory Distress Syndrome Receiving Extracorporeal Membrane Oxygenation: The Multicenter ECMObesity Study. Am. J. Respir. Crit. Care Med..

[102] Chang K., Li Y., Qin Z., Zhang Z., Wang L., Yang Q., Geng J., Deng N., Chen S., Su B. (2023). Effect of extracorporeal hemoadsorption in critically ill patients with COVID-19: A narrative review. Front. Immunol..

[103] Supady A., Weber E., Rieder M., Lother A., Niklaus T., Zahn T., Frech F., Müller S., Kuhl M., Benk C. (2021). Cytokine adsorption in patients with severe COVID-19 pneumonia requiring extracorporeal membrane oxygenation (CYCOV): A single centre, open-label, randomised, controlled trial. Lancet Respir. Med..

[104] Supady A., Brodie D., Wengenmayer T. (2022). Extracorporeal haemoadsorption: Does the evidence support its routine use in critical care?. Lancet Respir. Med..

[105] Ohshimo S. (2021). Oxygen administration for patients with ARDS. J. Intensive Care.

[106] Akella P., Voigt L.P., Chawla S. (2022). To Wean or Not to Wean: A Practical Patient Focused Guide to Ventilator Weaning. J. Intensive Care Med..

[107] Dorado J.H., Pérez J., Accoce M., Navarro E., Gilgado D.I., Cardoso G.P., Telias I., Brochard L.J. (2023). Oxygenation or Driving Pressure for Setting PEEP in Obese Patients With COVID-19 ARDS. Respir. Care.

[108] Bhimraj A., Morgan R.L., Shumaker A.H., Baden L.R., Cheng V.C.-C., Edwards K.M., Gallagher J.C., Gandhi R.T., Muller W.J., Nakamura M.M. (2022). Infectious Diseases Society of America Guidelines on the Treatment and Management of Patients with COVID-19 (September 2022). Clin. Infect. Dis..

[109] Pereira L.F., Dallagnol C.A., Moulepes T.H., Hirota C.Y., Kutsmi P., dos Santos L.V., Pirich C.L., Picheth G.F. (2023). Oxygen therapy alternatives in COVID19: From classical to nanomedicine. Heliyon.

[110] Chatterjee B., Thakur S.S. (2022). Remdesivir and Its Combination With Repurposed Drugs as COVID-19 Therapeutics. Front. Immunol..

[111] Li G., Hilgenfeld R., Whitley R., De Clercq E. (2023). Therapeutic strategies for COVID-19: Progress and lessons learned. Nat. Rev. Drug Discov..

[112] Thiruchelvam K., Kow C.S., Hadi M.A., Hasan S.S. (2022). The use of remdesivir for the management of patients with moderate-to-severe COVID-19: A systematic review. Expert Rev. Anti-Infect. Ther..

[113] Cocuz M.-E., Cocuz I.G., Rodina L., Tataranu E., Caliman-Sturdza O.A., Filip F. (2024). Treatment with Remdesivir of Children with SARS-CoV-2 Infection: Experience from a Clinical Hospital in Romania. Life.

[114] Shabbir B., Malik U., Sarfraz Z., Saeed F., Nawaz K., Khalid I., Gondal K.M. (2024). Efficacy of Remdesivir on Clinical Outcomes in COVID-19 Patients: A Study in a Tertiary Care Hospital in Pakistan. J. Community Hosp. Intern. Med. Perspect..

[115] Akinosoglou K., Rigopoulos E.A., Schinas G., Kaiafa G., Polyzou E., Tsoupra S., Tzouvelekis A., Gogos C., Savopoulos C. (2023). Remdesivir Use in the Real-World Setting: An Overview of Available Evidence. Viruses.

[116] Mozaffari E., Chandak A., Gottlieb R.L., Chima-Melton C., Berry M., Amin A.N., Sax P.E., Kalil A.C. (2024). Remdesivir-Associated Survival Outcomes Among Immunocompromised Patients Hospitalized for COVID-19: Real-world Evidence From the Omicron-Dominant Era. Clin. Infect. Dis..

[117] Batool S., Chokkakula S., Jeong J.H., Baek Y.H., Song M.S. (2025). SARS-CoV-2 drug resistance and therapeutic approaches. Heliyon.

[118] Study Details|Study of Obeldesivir in Participants With COVID-19 Who Have a High Risk of Developing Serious or Severe Illness|ClinicalTrials.gov n.d.. https://clinicaltrials.gov/study/NCT05603143?cond=NCT05603143&rank=1.

[119] Gounden V., Bhatt H., Jialal I. (2025). Renal Function Tests. StatPearls [Internet].

[120] FakhriRavari A., Malakouti M. (2024). Remdesivir and the Liver: A Concise Narrative Review of Remdesivir-Associated Hepatotoxicity in Patients Hospitalized Due to COVID-19. Pharmacoepidemiology.

[121] Hashemian S.M.R., Sheida A., Taghizadieh M., Memar M.Y., Hamblin M.R., Baghi H.B., Nahand J.S., Asemi Z., Mirzaei H. (2023). Paxlovid (Nirmatrelvir/Ritonavir): A new approach to COVID-19 therapy?. Biomed. Pharmacother..

[122] Hammond J., Leister-Tebbe H., Gardner A., Abreu P., Bao W., Wisemandle W., Baniecki M., Hendrick V.M., Damle B., Simón-Campos A. (2022). Oral Nirmatrelvir for High-Risk, Nonhospitalized Adults with COVID-19. N. Engl. J. Med..

[123] Takashita E., Kinoshita N., Yamayoshi S., Sakai-Tagawa Y., Fujisaki S., Ito M., Iwatsuki-Horimoto K., Halfmann P., Watanabe S., Maeda K. (2022). Efficacy of Antiviral Agents against the SARS-CoV-2 Omicron Subvariant BA.2. N. Engl. J. Med..

[124] Imai M., Ito M., Kiso M., Yamayoshi S., Uraki R., Fukushi S., Watanabe S., Suzuki T., Maeda K., Sakai-Tagawa Y. (2023). Efficacy of Antiviral Agents against Omicron Subvariants BQ.1.1 and XBB. N. Engl. J. Med..

[125] Tamura T.J., Choudhary M.C., Deo R., Yousuf F., Gomez A.N., Edelstein G.E., Boucau J., Glover O.T., Barry M., Gilbert R.F. (2024). Emerging SARS-CoV-2 Resistance After Antiviral Treatment. JAMA Netw. Open.

[126] Sanderson T., Hisner R., Donovan-Banfield I., Hartman H., Løchen A., Peacock T.P., Ruis C. (2023). A molnupiravir-associated mutational signature in global SARS-CoV-2 genomes. Nature.

[127] Fountain-Jones N.M., Vanhaeften R., Williamson J., Maskell J., Chua I.-L.J., Charleston M., Cooley L. (2024). Effect of molnupiravir on SARS-CoV-2 evolution in immunocompromised patients: A retrospective observational study. Lancet Microbe.

[128] Lee C.-C., Hsieh C.-C., Ko W.-C. (2021). Molnupiravir—A Novel Oral Anti-SARS-CoV-2 Agent. Antibiotics.

[129] FDA Approves Veklury Remdesivir for COVID 19 Treatment in Patients With Severe Renal Impairment Including Those on Dialysis n.d.. https://www.gilead.com/news/news-details/2023/fda-approves-veklury-remdesivir-for-covid-19-treatment-in-patients-with-severe-renal-impairment-including-those-on-dialysis.

[130] Reis G., Silva E.A.M., Silva D.C.M., Thabane L., Campos V.H.S., Ferreira T.S., Santos C.V., Nogueira A.M., Almeida A.P., Savassi L.C. (2023). Early Treatment with Pegylated Interferon Lambda for COVID-19. N. Engl. J. Med..

[131] Shen Y., Ai J., Lin N., Zhang H., Li Y., Wang H., Wang S., Wang Z., Li T., Sun F. (2022). An open, prospective cohort study of VV116 in Chinese participants infected with SARS-CoV-2 omicron variants. Emerg. Microbes Infect..

[132] Yotsuyanagi H., Ohmagari N., Doi Y., Yamato M., Fukushi A., Imamura T., Sakaguchi H., Sonoyama T., Sanaki T., Ichihashi G. (2024). Prevention of post COVID-19 condition by early treatment with ensitrelvir in the phase 3 SCORPIO-SR trial. Antivir. Res..

[133] Yotsuyanagi H., Ohmagari N., Doi Y., Yamato M., Bac N.H., Cha B.K., Imamura T., Sonoyama T., Ichihashi G., Sanaki T. (2024). Efficacy and Safety of 5-Day Oral Ensitrelvir for Patients With Mild to Moderate COVID-19. JAMA Netw. Open.

[134] Xocova® (Ensitrelvir Fumaric Acid) Tablets 125mg Approved in Japan for the Treatment of SARS-CoV-2 Infection, Under the Emergency Regulatory Approval System | News | Shionogi Co., Ltd. n.d.. https://www.shionogi.com/global/en/news/2022/11/e20221122.html.

[135] Barnette K.G., Gordon M.S., Rodriguez D., Bird T.G., Skolnick A., Schnaus M., Skarda P.K., Lobo S., Sprinz E., Arabadzhiev G. (2022). Oral Sabizabulin for High-Risk, Hospitalized Adults with COVID-19: Interim Analysis. NEJM Evid..

[136] Mortezavi M., Sloan A., Singh R.S.P., Chen L.F., Kim J.H., Shojaee N., Toussi S.S., Prybylski J., Baniecki M.L., Bergman A. (2025). Virologic Response and Safety of Ibuzatrelvir, A Novel SARS-CoV-2 Antiviral, in Adults With COVID-19. Clin. Infect. Dis..

[137] Liu Z., Shi F., Liu J.-X., Liu J.-Q., Li J., Wang Q., Wang H., Gao C.-L., Li J.-M., Zhao D.-F. (2021). Clinical Efficacy of Corticosteroids in the Early Stages of Deterioration in COVID-19 Pneumonia. Infect. Drug Resist..

[138] Shamim L., Musharaf I., Nashwan A.J. (2025). Dexamethasone in coronavirus disease 2019 care: Dosage and utilization insights. World J. Virol..

[139] El Mezzeoui S., El Aidouni G., Merbouh M., El Kaouini A., Aftiss F.Z., Berrichi S., Berrajaa S., Bkiyer H., Abda N., Housni B. (2021). Dexamethasone or methylprednisolone therapy in COVID-19 pneumonia: A retrospective and comparative study of 513 cases. Ann. Med. Surg..

[140] The RECOVERY Collaborative Group (2021). Dexamethasone in Hospitalized Patients with COVID-19. N. Engl. J. Med..

[141] Lipworth B.J., Chan R., Kuo C.R. (2020). Tocilizumab for severe COVID-19 pneumonia. Lancet Rheumatol..

[142] Edinoff A.N., Alpaugh E.S., Newgaard O., Wajid I., Klapper R.J., Cornett E.M., Kaye A.M., Iyer P., Kaye A.D. (2023). Tocilizumab for Severe COVID-19 Infection and Multisystem Inflammatory Syndrome in Adults and Children. Life.

[143] Mao Y., Guo A., Zhang Y., Lai J., Yuan D., Zhang H., Diao W., Chen W., Yan F. (2025). Baricitinib treatment for hospitalized patients with severe COVID-19 on invasive mechanical ventilation: A propensity score-matched and retrospective analysis. Front. Med..

[144] Balkhair A., Al-Zakwani I., Al Busaidi M., Al-Khirbash A., Al Mubaihsi S., BaTaher H., Al Aghbari J., Al Busaidi I., Al Kindi M., Baawain S. (2021). Anakinra in hospitalized patients with severe COVID-19 pneumonia requiring oxygen therapy: Results of a prospective, open-label, interventional study. Int. J. Infect. Dis..

[145] Taylor P.C., Adams A.C., Hufford M.M., de la Torre I., Winthrop K., Gottlieb R.L. (2021). Neutralizing monoclonal antibodies for treatment of COVID-19. Nat. Rev. Immunol..

[146] Cugno M., Meroni P.L., Gualtierotti R., Griffini S., Grovetti E., Torri A., Panigada M., Aliberti S., Blasi F., Tedesco F. (2020). Complement activation in patients with COVID-19: A novel therapeutic target. J. Allergy Clin. Immunol..

[147] Carvelli J., Piperoglou C., Farnarier C., Vely F., Mazodier K., Audonnet S., Nitschke P., Bole-Feysot C., Boucekine M., Cambon A. (2020). Functional and genetic testing in adults with HLH reveals an inflammatory profile rather than a cytotoxicity defect. Blood.

[148] Benmansour N.C., Carvelli J., Vivier E. (2021). Complement cascade in severe forms of COVID-19: Recent advances in therapy. Eur. J. Immunol..

[149] Gordon A.C., Mouncey P.R., Al-Beidh F., Rowan K.M., Nichol A.D., Arabi Y.M., Annane D., Beane A., Van Bentum-Puijk W., Berry L.R. (2021). Interleukin-6 Receptor Antagonists in Critically Ill Patients with COVID-19. N. Engl. J. Med..

[150] Hermine O., Mariette X., Tharaux P.-L., Resche-Rigon M., Porcher R., Ravaud P., Bureau S., Dougados M., Tibi A., CORIMUNO-19 Collaborative Group (2021). Effect of Tocilizumab vs Usual Care in Adults Hospitalized With COVID-19 and Moderate or Severe Pneumonia. JAMA Intern. Med..

[151] Salama C., Han J., Yau L., Reiss W.G., Kramer B., Neidhart J.D., Criner G.J., Kaplan-Lewis E., Baden R., Pandit L. (2021). Tocilizumab in Patients Hospitalized with COVID-19 Pneumonia. N. Engl. J. Med..

[152] Salvarani C., Dolci G., Massari M., Merlo D.F., Cavuto S., Savoldi L., Bruzzi P., Boni F., Braglia L., Turrà C. (2021). Effect of Tocilizumab vs Standard Care on Clinical Worsening in Patients Hospitalized With COVID-19 Pneumonia. JAMA Intern. Med..

[153] Veiga V.C., Prats J.A.G.G., Farias D.L.C., Rosa R.G., Dourado L.K., Zampieri F.G., Machado F.R., Lopes R.D., Berwanger O., Azevedo L.C.P. (2021). Effect of tocilizumab on clinical outcomes at 15 days in patients with severe or critical coronavirus disease 2019: Randomised controlled trial. BMJ.

[154] RECOVERY Collaborative Group (2021). Tocilizumab in patients admitted to hospital with COVID-19 (RECOVERY): A randomised, controlled, open-label, platform trial. Lancet.

[155] Wang Y., Hao A., Ji P., Ma Y., Zhang Z., Chen J., Mao Q., Xiong X., Rehati P., Wang Y. (2024). A bispecific antibody exhibits broad neutralization against SARS-CoV-2 Omicron variants XBB.1.16, BQ.1.1 and sarbecoviruses. Nat. Commun..

[156] Center for Biologics Evaluation and Research (CBER)|FDA n.d.. https://www.fda.gov/about-fda/fda-organization/center-biologics-evaluation-and-research-cber.

[157] Pickens C.O., Gao C.A., Cuttica M.J., Smith S.B., Pesce L.L., Grant R.A., Kang M., Morales-Nebreda L., Bavishi A.A., Arnold J.M. (2021). Bacterial Superinfection Pneumonia in Patients Mechanically Ventilated for COVID-19 Pneumonia. Am. J. Respir. Crit. Care Med..

[158] Langford B.J., So M., Raybardhan S., Leung V., Soucy J.-P.R., Westwood D., Daneman N., MacFadden D.R. (2021). Antibiotic prescribing in patients with COVID-19: Rapid review and meta-analysis. Clin. Microbiol. Infect..

[159] Zayet S., Klopfenstein T. (2022). Antibiotics and Therapeutic Agent Prescription in COVID-19 Management. Antibiotics.

[160] Hoenigl M., Seidel D., Sprute R., Cunha C., Oliverio M., Goldman G.H., Ibrahim A.S., Carvalho A. (2022). COVID-19-associated fungal infections. Nat. Microbiol..

[161] Gezer D., Yurtsever S., Ülger S.T. (2025). Bacterial and Fungal Infections Among COVID-19 Patients in Intensive Care Unit. Med. Rec..

[162] Rovina N., Koukaki E., Romanou V., Ampelioti S., Loverdos K., Chantziara V., Koutsoukou A., Dimopoulos G. (2022). Fungal Infections in Critically Ill COVID-19 Patients: Inevitabile Malum. J. Clin. Med..

[163] Dimopoulos G., Almyroudi M.-P., Myrianthefs P., Rello J. (2021). COVID-19-Associated Pulmonary Aspergillosis (CAPA). J. Intensive Med..

[164] Theodosiou A.A., Jones C.E., Read R.C., Bogaert D. (2023). Microbiotoxicity: Antibiotic usage and its unintended harm to the microbiome. Curr. Opin. Infect. Dis..

[165] Nafea A.M., Wang Y., Wang D., Salama A.M., Aziz M.A., Xu S., Tong Y. (2024). Application of next-generation sequencing to identify different pathogens. Front. Microbiol..

[166] Candel F.J., Salavert M., Cantón R., del Pozo J.L., Galán-Sánchez F., Navarro D., Rodríguez A., Rodríguez J.C., Rodríguez-Aguirregabiria M., Suberviola B. (2024). The role of rapid multiplex molecular syndromic panels in the clinical management of infections in critically ill patients: An experts-opinion document. Crit. Care.

[167] Fragkou P.C., Palaiodimou L., Stefanou M.I., Katsanos A.H., Lambadiari V., Paraskevis D., Andreadou E., Dimopoulou D., Zompola C., Ferentinos P. (2022). Effects of low molecular weight heparin and fondaparinux on mortality, hemorrhagic and thrombotic complications in COVID-19 patients. Ther. Adv. Neurol. Disord..

[168] Bikdeli B., Madhavan M.V., Jimenez D., Chuich T., Dreyfus I., Driggin E., Nigoghossian C.D., Ageno W., Madjid M., Guo Y. (2020). COVID-19 and Thrombotic or Thromboembolic Disease: Implications for Prevention, Antithrombotic Therapy, and Follow-Up. J. Am. Coll. Cardiol..

[169] Bradbury C.A., McQuilten Z. (2022). Anticoagulation in COVID-19. Lancet.

[170] Zhao R., Su Z., Komissarov A.A., Liu S.-L., Yi G., Idell S., Matthay M.A., Ji H.-L. (2021). Associations of D-Dimer on Admission and Clinical Features of COVID-19 Patients: A Systematic Review, Meta-Analysis, and Meta-Regression. Front. Immunol..

[171] Goswami J., MacArthur T.A., Sridharan M., Pruthi R.K., McBane R.D., Witzig T.E., Park M.S. (2021). A Review of Pathophysiology, Clinical Features, and Management Options of COVID-19 Associated Coagulopathy. Shock.

[172] Makarem A., Zareef R., Abourjeili J., Nassar J.E., Bitar F., Arabi M. (2023). Low molecular weight heparin in COVID-19: Benefits and concerns. Front. Pharmacol..

[173] Rahi M.S., Jindal V., Reyes S.-P., Gunasekaran K., Gupta R., Jaiyesimi I. (2021). Hematologic disorders associated with COVID-19: A review. Ann. Hematol..

[174] Ragnoli B., Da Re B., Galantino A., Kette S., Salotti A., Malerba M. (2023). Interrelationship between COVID-19 and Coagulopathy: Pathophysiological and Clinical Evidence. Int. J. Mol. Sci..

[175] Braz-de-Melo H.A., Faria S.S., Pasquarelli-do-Nascimento G., Santos I.D.O., Kobinger G.P., Magalhães K.G. (2021). The Use of the Anticoagulant Heparin and Corticosteroid Dexamethasone as Prominent Treatments for COVID-19. Front. Med..

[176] Xu S., Ilyas I., Weng J. (2023). Endothelial dysfunction in COVID-19: An overview of evidence, biomarkers, mechanisms and potential therapies. Acta Pharmacol. Sin..

[177] Kerna N.A., Flores J.V., Pruitt K.D., Carsrud N.D.V., Ngwu D.C., Rodriguez D., Holets H.M., Nwokorie U., Jomsky B.M., Senat A.J.B. (2024). Hematological Conditions Associated with COVID-19: Pathophysiology, Clinical Manifestations, and Therapeutic Approaches. Eur. J. Med. Health Res..

[178] Nicolai L., Leunig A., Brambs S., Kaiser R., Weinberger T., Weigand M., Muenchhoff M., Hellmuth J.C., Ledderose S., Schulz H. (2020). Immunothrombotic Dysregulation in COVID-19 Pneumonia Is Associated With Respiratory Failure and Coagulopathy. Circulation.

[179] Pfortmueller C.A., Spinetti T., Urman R.D., Luedi M.M., Schefold J.C. (2021). COVID-19-associated acute respiratory distress syndrome (CARDS): Current knowledge on pathophysiology and ICU treatment—A narrative review. Best Pr. Res. Clin. Anaesthesiol..

[180] Wu T., Zuo Z., Kang S., Jiang L., Luo X., Xia Z., Liu J., Xiao X., Ye M., Deng M. (2020). Multi-organ Dysfunction in Patients with COVID-19: A Systematic Review and Meta-analysis. Aging Dis..

[181] Keswani M., Mehta N., Mazer-Amirshahi M., Tran Q.K., Pourmand A. (2022). Sedation in mechanically ventilated COVID-19 patients: A narrative review for emergency medicine providers. Am. J. Emerg. Med..

[182] Kubde D., Badge A.K., Ugemuge S., Shahu S. (2023). Importance of Hospital Infection Control. Cureus.

[183] Ölmez H., Tosun M. (2023). Significance of laboratory biomarkers in monitoring patients with COVID-19 pneumonia. Health Sci. Q..

[184] Nalbandian A., Sehgal K., Gupta A., Madhavan M.V., McGroder C., Stevens J.S., Cook J.R., Nordvig A.S., Shalev D., Sehrawat T.S. (2021). Post-acute COVID-19 syndrome. Nat. Med..

[185] Zhang H., Weng Z., Zheng Y., Zheng M., Chen W., He H., Ye X., Zheng Y., Xie J., Zheng K. (2023). Epidemiological and clinical features of SARS-CoV-2 Omicron variant infection in Quanzhou, Fujian province: A retrospective study. Sci. Rep..

[186] Chatterjee S., Bhattacharya M., Nag S., Dhama K., Chakraborty C. (2023). A Detailed Overview of SARS-CoV-2 Omicron: Its Sub-Variants, Mutations and Pathophysiology, Clinical Characteristics, Immunological Landscape, Immune Escape, and Therapies. Viruses.

[187] Qu P., Faraone J.N., Evans J.P., Zheng Y.M., Carlin C., Anghelina M., Stevens P., Fernandez S., Jones D., Panchal A.R. (2023). Enhanced evasion of neutralizing antibody response by Omicron XBB.1.5, CH.1.1, and CA.3.1 variants. Cell Rep..

[188] Lasrado N., Collier A.-R.Y., Hachmann N.P., Miller J., Rowe M., Schonberg E.D., Rodrigues S.L., LaPiana A., Patio R.C., Anand T. (2023). Neutralization escape by SARS-CoV-2 Omicron subvariant BA.2.86. Vaccine.

[189] Hoteit R., Yassine H.M. (2022). Biological Properties of SARS-CoV-2 Variants: Epidemiological Impact and Clinical Consequences. Vaccines.

[190] Markov P.V., Ghafari M., Beer M., Lythgoe K., Simmonds P., Stilianakis N.I., Katzourakis A. (2023). The evolution of SARS-CoV-2. Nat. Rev. Microbiol..

[191] Chung Y.S., Lam C.Y., Tan P.H., Tsang H.F., Wong S.C.C. (2024). Comprehensive Review of COVID-19: Epidemiology, Pathogenesis, Advancement in Diagnostic and Detection Techniques, and Post-Pandemic Treatment Strategies. Int. J. Mol. Sci..

[192] Kainov D.E., Ravlo E., Ianevski A. (2025). Seeking innovative concepts in development of antiviral drug combinations. Antivir. Res..

[193] Levy M.E., Chilunda V., Davis R.E., Heaton P.R., Pawloski P.A., Goldman J.D., Schandl C.A., McEwen L.M., Cirulli E.T., Wyman D. (2024). Reduced Likelihood of Hospitalization With the JN.1 or HV.1 Severe Acute Respiratory Syndrome Coronavirus 2 Variants Compared With the EG.5 Variant. J. Infect. Dis..

[194] Feng B., Fu K. (2023). Latest development of approved COVID-19 drugs and COVID-19 drugs undergoing late stage clinical trials. Front. Drug Discov..

[195] Giannitsioti E., Mavroudis P., Speggos I., Katsoulidou A., Pantazis N., Loupis T., Daniil I., Rekleiti N., Damianidou S., Louka C. (2023). Real life treatment experience and outcome of consecutively hospitalised patients with SARS-CoV-2 pneumonia by Omicron-1 vs Delta variants. Infect. Dis..

[196] Meyerowitz E.A., Li Y. (2024). Review: The Landscape of Antiviral Therapy for COVID-19 in the Era of Widespread Population Immunity and Omicron-Lineage Viruses. Clin. Infect. Dis..

[197] Wong J.Y., Cheung J.K., Lin Y., Bond H.S., Lau E.H.Y., Ip D.K.M., Cowling B.J., Wu P. (2023). Intrinsic and Effective Severity of Coronavirus Disease 2019 Cases Infected With the Ancestral Strain and Omicron BA.2 Variant in Hong Kong. J. Infect. Dis..

[198] Wafy S.M., Saman M.O., Ahmed M.K. (2024). Predictors of mortality of hospitalized COVID-19 pneumonia patients in university hospital. Egypt. J. Bronchol..

[199] Khurana A.K., Hussain A., Goyal A., Karna S.T., Care A.A.C., Saigal S., Soman R.K., Verma M., Khadanga S., Sahu G. (2022). Six-Week Hospital-Based Pulmonary Rehabilitation in Covid Pneumonia ICU Survivors: Experience from a Tertiary Care Center in Central India. Turk. Thorac. J..

[200] Hendrickson K.W., Peltan I.D., Brown S.M. (2021). The Epidemiology of Acute Respiratory Distress Syndrome Before and After Coronavirus Disease 2019. Crit. Care Clin..

[201] Kuzmin B., Movsisyan A., Praetsch F., Schilling T., Lux A., Fadel M., Azizzadeh F., Crackau J., Keyser O., Awad G. (2023). Outcomes of patients with coronavirus disease versus other lung infections requiring venovenous extracorporeal membrane oxygenation. Heliyon.

[202] Klimkiewicz J., Grzywacz A., Michałowski A., Gutowski M., Paryż K., Jędrych E., Lubas A. (2024). Acute Kidney Injury and Chronic Kidney Disease and Their Impacts on Prognosis among Patients with Severe COVID-19 Pneumonia: An Expert Center Case–Cohort Study. J. Clin. Med..

[203] Martínez E., Aguilera C., Márquez D., Ziegler G., Plumet J., Tschopp L., Cominotti C., Sturzenegger V., Cimino C., Escobar H. (2023). Post intensive care syndrome in survivors of COVID-19 who required mechanical ventilation during the third wave of the pandemic: A prospective study. Hear. Lung.

[204] Maison D.P., Tasissa H., Deitchman A., Peluso M.J., Deng Y., Miller F.D., Henrich T.J., Gerschenson M. (2025). COVID-19 clinical presentation, management, and epidemiology: A concise compendium. Front. Public Health.

[205] Alkhalifa H.A., Darwish E., Alsalman Z., Alfaraj A., Alkhars A., Alkhalifa F., Algaraash M., Elshebiny A.M., Alkhoufi E., Elzorkany K.M.A. (2025). Predictors of developing severe COVID-19 among hospitalized patients: A retrospective study. Front. Med..

[206] DeWolf S., Laracy J.C., Perales M.-A., Kamboj M., van den Brink M.R.M., Vardhana S. (2022). SARS-CoV-2 in immunocompromised individuals. Immunity.

[207] Rahmati M., Shamsi M.M., Khoramipour K., Malakoutinia F., Woo W., Park S., Yon D.K., Lee S.W., Shin J.I., Smith L. (2022). Baseline physical activity is associated with reduced mortality and disease outcomes in COVID-19: A systematic review and meta-analysis. Rev. Med. Virol..

[208] Kumar M., Baig M.S., Bhardwaj K. (2025). Advancements in the development of antivirals against SARS-Coronavirus. Front. Cell. Infect. Microbiol..

[209] Diamond M., Peniston H.L., Sanghavi D.K., Mahapatra S. (2025). Acute Respiratory Distress Syndrome. StatPearls.

[210] Meawed T.E., Ahmed S.M., Mowafy S.M., Samir G.M., Anis R.H. (2021). Bacterial and fungal ventilator associated pneumonia in critically ill COVID-19 patients during the second wave. J. Infect. Public Health.

[211] Poggiali E., Bastoni D., Ioannilli E., Vercelli A., Magnacavallo A. (2020). Deep Vein Thrombosis and Pulmonary Embolism: Two Complications of COVID-19 Pneumonia?. Eur. J. Case Rep. Intern. Med..

[212] Shao H.-H., Yin R.-X. (2024). Pathogenic mechanisms of cardiovascular damage in COVID-19. Mol. Med..

[213] Mirouse A., Friol A., Moreau A.-S., Jung B., Jullien E., Bureau C., Djibré M., de Prost N., Zafrani L., Argaud L. (2023). Severe SARS-Cov2 pneumonia in vaccinated patients: A multicenter cohort study. Sci. Rep..

[214] Bogusławski S., Strzelak A., Gajko K., Peradzyńska J., Popielska J., Marczyńska M., Kulus M., Krenke K. (2023). The outcomes of COVID-19 pneumonia in children—Clinical, radiographic, and pulmonary function assessment. Pediatr. Pulmonol..

[215] Constantin T., Pék T., Horváth Z., Garan D., Szabó A.J. (2023). Multisystem inflammatory syndrome in children (MIS-C): Implications for long COVID. Inflammopharmacology.

[216] Sundeep H.K., Litty P., Nimmi K., Hozaifah M., Faek A.E.T., Bashir E.F.A., Amar H. (2020). Clinical characteristics, management, maternal and neonatal outcome among seven severe and critically ill pregnant women with COVID-19 pneumonia. Clin. J. Obstet. Gynecol..

[217] Zampogna E., Paneroni M., Belli S., Aliani M., Gandolfo A., Visca D., Bellanti M.T., Ambrosino N., Vitacca M. (2021). Pulmonary Rehabilitation in Patients Recovering from COVID-19. Respiration.

[218] Gu X., Zheng M., Gao Y., Lin S., Zhang X., Chen C., Zhu H., Sun W., Zhang Y. (2025). Overview of host-directed antiviral targets for future research and drug development. Acta Pharm. Sin. B.

